# The bile salt deoxycholate induces *Campylobacter jejuni* genetic point mutations that promote increased antibiotic resistance and fitness

**DOI:** 10.3389/fmicb.2022.1062464

**Published:** 2022-12-21

**Authors:** Prabhat K. Talukdar, Torin M. Crockett, Lisa M. Gloss, Steven Huynh, Steven A. Roberts, Kyrah L. Turner, Sebastien T. E. Lewis, Tristin L. Herup-Wheeler, Craig T. Parker, Michael E. Konkel

**Affiliations:** ^1^School of Molecular Biosciences, College of Veterinary Medicine, Washington State University, Pullman, WA, United States; ^2^Produce Safety and Microbiology, United States Department of Agriculture-Agricultural Research Service, Albany, CA, United States

**Keywords:** sodium deoxycholate, reactive oxygen species, fluoroquinolone resistance, DNA lesions, DNA repair

## Abstract

Oxidative damage to DNA is a significant source of mutations in living organisms. While DNA damage must be repaired to maintain the integrity of the genome and cell survival, errors made during DNA repair may contribute to evolution. Previous work has revealed that *Campylobacter jejuni* growth in the presence of bile salt deoxycholate (DOC) causes an increase in reactive oxygen species and the occurrence of 8-oxo-deoxyguanosine (8-oxo-dG) DNA lesions. The fundamental goal of this project was to determine if *C. jejuni* growth in a medium containing DOC contributes to DNA mutations that provide a fitness advantage to the bacterium. Co-culture experiments revealed that *C. jejuni* growth in a DOC-supplemented medium increases the total number of ciprofloxacin-resistant isolates compared to *C. jejuni* grown in the absence of DOC. We recovered two individual isolates grown in a medium with DOC that had a point mutation in the gene encoding the EptC phosphoethanolamine transferase. Transformants harboring the EptC variant protein showed enhanced resistance to the antimicrobial agent polymyxin B and DOC when compared to an *eptC* deletion mutant or the isolate complemented with a wild-type copy of the gene. Finally, we found that the base excision repair (BER), homologous recombination repair (HRR), and nucleotide excision repair (NER) are involved in general oxidative damage repair in *C. jejuni* but that the BER pathway plays the primary role in the repair of the 8-oxo-dG lesion. We postulate that bile salts drive *C. jejuni* mutations (adaptations) and enhance bacterial fitness in animals.

## Introduction

Bacteria encounter bile salts inside the intestines of warm-blooded animals. The concentration of bile salts ranges from 0.2% to 20% in the intestines of humans (Elliot, 1985), 0.7% in the jejunum, and 0.01% in the cecum of chickens (Lin et al., 2003). Bile salts are primarily composed of salts of cholic, deoxycholic, chenodeoxycholic, and lithocholic acids. Deoxycholic acid (DOC), a secondary bile salt, constitutes 15% of the total bile salts. Bacteria have intricate relations with intestinal bile salts. The composition of bile salts is shaped by the metabolic activities of the intestinal anaerobes (Ridlon et al., 2006). In turn, bile salts dictate the composition of intestinal microbiota (Kakiyama et al., 2013) and act as a protective agent against pathogenic microbes due to their antimicrobial activities (Hofmann and Eckmann, 2006; Sannasiddappa et al., 2017). Bile salts disrupt bacterial membranes, denature proteins, and cause DNA lesions in the bacterial genome (Merritt and Donaldson, 2009; Urdaneta and Casadesus, 2017). Intestinal bacteria adapt to the detrimental effect of bile salts through different physiological changes, including the remodeling of the cell envelope and activation of efflux and stress response systems (Hernandez et al., 2012). *Campylobacter jejuni*, a Gram-negative microaerophilic bacterium, is exposed to bile within the gut of vertebrate hosts.

*Campylobacter* species are the most common culture-proven cause of bacterial gastroenteritis worldwide, accounting for 400–500 million cases of diarrhea each year (Ruiz-Palacios, 2007). In developed countries, infection with certain strains of *C. jejuni* has been correlated with a higher incidence of Guillain-Barré syndrome (GBS), reactive arthritis, and/or post infectious-irritable bowel syndrome (PI-IBS; Spiller et al., 2000; Allos, 2001; Schwerer, 2002; Frenzen, 2008; Sung et al., 2013). In low- and middle-income countries (LMIC), *C. jejuni* infections result in malnourishment and stunted growth (Haque et al., 2019). Alarmingly, reports of antibiotic-resistant *Campylobacter* are increasing (Engberg et al., 2001; Coker et al., 2002; Bae et al., 2005; Gibreel and Taylor, 2006; Ruiz-Palacios, 2007). In a recent report, ~40% of *Campylobacter* isolates recovered from humans were fluoroquinolone (FQ)-resistant (Bravo et al., 2021). FQ-resistant *Campylobacter* spp. have been added to the World Health Organization’s (WHO) priority list of antibiotic-resistant bacteria (Tacconelli et al., 2018).

Several mechanisms of resistance to FQs have been reported in Gram-negative bacteria, including mutations in the genes encoding the topoisomerase enzymes [topoisomerase II (DNA gyrase, composed of two subunits of GyrA and two subunits of GyrB) and topoisomerase IV (ParC and ParE)], decreased outer membrane permeability, and export through an active efflux system (Payot et al., 2006). In *Campylobacter* spp., studies have mainly focused on the determination of *gyrA* mutations conferring resistance, as the *parC*/*parE* appear to be absent in the genomes of sequenced *C. jejuni* isolates (Luo et al., 2003; Luo et al., 2005; Payot et al., 2006). *C. jejuni* isolates resistant to FQs frequently have mutations in DNA gyrase, particularly in the quinolone resistance determining region (QRDR) of the GyrA. DNA gyrase is responsible for introducing negative supercoils into DNA to relieve the topological stress from transcription and replication complexes (Hawkey, 2003). The Thr-86-Ile mutation is the most common cause of class-wide FQ resistance among *C. jejuni* isolates and confers a high-level FQ resistance (Hakanen et al., 2002; Beckmann et al., 2004). Other less common mutations have been reported in GyrA (i.e., Thr-86-Ala, Thr-86-Lys, Asp-90-Asn, and Asp-90-Tyr; Changkwanyeun et al., 2016). Poultry has been and continues to be a significant source of FQ-resistant *Campylobacter*, perhaps due to the past practice of using FQs in food animal production. Even though FQs are no longer used in poultry production, FQ-resistant *Campylobacter* organisms have persisted and even increased (Price et al., 2007; Sproston et al., 2018). Previous work has revealed that a single point mutation (i.e., Thr-86-Ile) in *gyrA*, which confers high-level of resistance to FQs, provides *Campylobacter* bacteria with a fitness advantage in which the FQ-resistant isolate outcompetes the FQ-susceptible isolate in chickens (Luo et al., 2005).

The ability of a pathogen to acquire mutations is essential for survival in damaging environments and is closely connected to the mechanisms of prokaryotic DNA repair systems. *C. jejuni* possesses DNA repair systems that function in base excision repair (BER), homologous recombination repair (HRR), and nucleotide excision repair (NER). Repair of double-strand breaks in *C. jejuni* occurs *via* the AddAB proteins (functionally analogous to the HRR proteins and RecBCD proteins of *Escherichia coli*) working with RecA (Gourley et al., 2017). BER repairs lesions that cause minor distortion in the DNA helix structure, such as those caused by oxidation, deamination, and alkylation (Krokan and Bjørås, 2013). In contrast, NER fixes damage from bulky DNA adducts, such as the pyrimidine dimers caused by UV light (Kisker et al., 2013). It is noteworthy that *C. jejuni* lacks the mutagenic/error-prone DNA polymerases (Pol II, Pol IV, and Pol V) that many bacteria use to adapt to environmental DNA-damaging agents.

*C. jejuni* passage through poultry and *C. jejuni* infections in humans are known to result in novel genotypic variants characterized by nucleotide polymorphisms and, depending on the strain, genomic rearrangements (Barton et al., 2007; Ridley et al., 2008). However, the mechanism(s) responsible for generating novel *C. jejuni* variants is currently not known. Previously, we reported that the bile salt DOC results in the production of reactive oxygen species (ROS) and causes DNA lesions in *C. jejuni* as determined by the increased production of 8-oxo-deoxyguanosine (8-oxo-dG) (Negretti et al., 2017). These DNA lesions could lead to genotypic variants unless they are repaired by the DNA repair system.

The types of DNA damage caused by ROS are typically repaired by BER, with the NER pathway acting as a secondary defense against oxidative damage (Demple and Harrison, 1994). The first step of the BER process involves DNA glycosylases removing the damaged nitrogenous base while leaving the sugar-phosphate backbone intact and creating an apurinic/apyrimidinic (AP) site. The 8-oxo-dG in many bacteria is removed by the DNA glycosylase MutM/Fpg (*aka* OGG1 in eukaryotes). If replication of the lesion yields an 8-oxo-dG: adenine base pair, a second DNA glycosylase termed MutY excises the adenine (*aka* hMYH in eukaryotes) to avoid making the mutation permanent in the next round of replication. *C. jejuni* possesses a MutY adenine DNA glycosylase but lacks a MutM/Fpg homolog. A previous study reported that a single nucleotide change in the *mutY* gene (a G ➔ T transversion at position 595) of *C. jejuni* results in an amazing ~100-fold higher frequency of ciprofloxacin resistance than the isogenic wild-type strain (Dai et al., 2015).

We hypothesized that *C. jejuni* growth in the presence of DOC in the gut causes mutations in the genome, some of which confer a fitness advantage in the intestine. The purpose of this study was, in part, to determine if *C. jejuni* growth in a medium containing the ROS-producing bile salt DOC causes DNA damage leading to adaptive mutations that can confer a fitness advantage. We sought to identify mutations, including those in genes and/or gene promoter regions, which could enhance the fitness of *C. jejuni* within an animal host and contribute to human disease. Our findings, together with data where multiple *C. jejuni* PFGE (pulsed-field gel electrophoresis)-type variants have been recovered from chickens inoculated with a single clone, support our hypothesis that stress-induced mutations are an important mechanism to improve fitness and increased virulence in *C. jejuni*.

## Materials and methods

### Bacterial strains and growth conditions

*C. jejuni* strain 81–176 was cultured on Mueller-Hinton (MH) agar containing 5% citrated bovine blood (MHB agar, Hardy Diagnostics, Santa Maria, CA) in a microaerobic atmosphere (5% O_2_, 10% CO_2_, 85% N_2_) at 37°C. The *C. jejuni* mutants and complemented isolates were generated as outlined below. When needed, MHB agar plates were supplemented with 8 μg/ml of chloramphenicol or 250 μg/ml of hygromycin. The isolates were passaged every 24–48 h on MHB agar. Sodium deoxycholate (Na-DOC) was used at a concentration of 0.05% unless it is stated otherwise. *E. coli* Stellar™ (Takara Bio Inc., Berkeley, CA) strain was cultured on LB-Miller agar or in LB-Miller broth (Fisher, Hampton, NH) under aerobic conditions at 37°C.

### Generation of ciprofloxacin-resistant isolates

*C. jejuni* were inoculated in MH broth and MH broth supplemented with 0.05% (w/v) Na-DOC at a final OD_540_ of 0.05 and placed on an orbital shaker at 225 rpm under microaerobic conditions at 37°C. The cultures were then passed into new flasks and normalized to the starting OD_540_ of 0.05, and the process was repeated for a total of 10 days. After 10-days, 10 ml of the cultures were centrifuged, serially diluted with sterile 1x PBS, and spread onto MHB agar plates supplemented with 0.125 μg/ml of ciprofloxacin. Once growth was observed on the MHB-ciprofloxacin plates, individual colonies were selected for PCR amplification and DNA sequencing to determine the mutations of the *gyrA* gene. This 10-day passage was repeated 4 more times for a total of 5 “runs.”

### Identifying point mutations in the *gyrA* gene

A fragment of the *gyrA* gene, containing the QRDR, was PCR amplified and sequenced. Individual bacterial colonies were suspended in water and boiled for 5 min. DNA fragments were amplified using CloneAmp™ HiFi PCR Premix (Takara Bio Inc., San Jose, CA) with the following parameters: 98°C for 4 min, 1 cycle; 98°C for 20 s, 55°C for 30 s, and 72°C for 1.5 min, 35 cycles; 72°C for 10 min, 1 cycle. The primers used in the PCR were gyrA-F and gyrA-R (Supplementary Table S1). PCR products were cleaned up by the GeneJET Gel Extraction Kit (Thermo Fisher Scientific, Vilnius, Lithuania). The *gyrA* gene sequence from each isolate was analyzed and compared to the sequence of *gyrA* from the 81–176 wild-type strain (ciprofloxacin-sensitive) using SnapGene software to identify DNA mutations at codons 86 and 90, which confer ciprofloxacin resistance to *C. jejuni*.

### Antimicrobial susceptibility tests

To determine the 50% inhibitory concentration (IC_50_) of ciprofloxacin, antimicrobial susceptibility tests were performed using the broth microdilution technique as described previously (Talukdar et al., 2021). The 96-well microtiter plate was incubated in an orbital shaker under microaerobic conditions at 37°C for 48 h. After incubation, the optical density was determined using a 96-well plate reader (BioTek Instruments Inc., Winooski, VT) at 595 nm (OD_595_). The concentrations and absorbance values were graphed on a logarithmic plot using GraphPad Prism (v6.0.h, La Jolla, CA), and the IC_50_ was calculated from the concentration-effect curve as described previously (Hakeem et al., 2019).

### Ciprofloxacin mutation frequency

To determine the mutation frequency of the *gyrA* gene, two cultures of the *C. jejuni* 81–176 wild-type strain were grown; one in a flask containing 50 ml of MH broth and the other in a flask with 50 ml MH broth with 0.05% Na-DOC and normalized to a starting OD_540_ of 0.04–0.05. The serial passage was continued for 5 days. After each day of serial passage, a portion of the Na-DOC-supplemented culture was transferred to a new flask of MH broth to minimize the variability in colony numbers following plating (a portion of the bacteria lysed in the presence of the detergent, Na-DOC). Following each day of incubation, 10 ml of each culture was centrifuged and resuspended in 1 ml of 1x PBS. Serial 10-fold dilutions were then performed with each sample, and 10 μl or 100 μl of each dilution was spread onto MHB agar plates without antibiotics and MHB agar plates with 8 μg/ml and 10 μg/ml ciprofloxacin. Once growth on MHB agar and MHB-ciprofloxacin plates could be observed, the colonies growing on each plate for both samples were counted, and the CFU/mL of ciprofloxacin-resistant (mutated) and total *C. jejuni* were determined for each sample. The mutation frequency was calculated by dividing the CFU/ml of *C. jejuni* ciprofloxacin-resistant isolates by the total CFU/ml. The assay was also performed with a *C. jejuni mutY* deletion mutant (∆*mutY*) and complemented isolate.

### Passage of *C. jejuni* in MH broth and MH DOC-supplemented medium

*C. jejuni* were inoculated in MH broth and MH broth supplemented with 0.05% Na-DOC at a final OD_540_ of 0.04 and incubated in a microaerobic chamber with shaking at 37°C. The cultures were passed into new flasks at an OD_540_ of 0.04–0.05 and the process was repeated until the ODs of the cultures in MH broth and MH DOC-supplemented medium were similar (MH-DOC adapted isolates) and the OD_540_ for the DOC-supplemented culture had stabilized (remained constant over several day periods). Thereafter, the cultures were inoculated into new flasks of MH broth and MH broth supplemented with 0.1% (w/v) Na-DOC at an OD_540_ of 0.05, and the process was repeated until the ODs of the cultures in MH broth and MH DOC-supplemented medium became similar (MH-DOC adapted isolates). The passage was repeated one additional time in MH broth and MH broth supplemented with 0.4% (w/v) Na-DOC. After each 24-h incubation period, a sample of the culture was plated on MHB agar plates containing 0.125 μg/ml of ciprofloxacin. To determine if the isolates had different sensitivities to DOC, individual colonies were selected and grown in MH broth containing 0.1% and 0.4% Na-DOC. The control consisted of the bacteria grown in MH broth without DOC.

### Genomic DNA extraction, whole genome sequencing, and analysis

Isolates were subjected to paired-end Illumina sequencing to accurately identify base substitutions, small insertions and deletions (indels), and copy number changes. Briefly, genomic DNA was extracted from *C. jejuni* isolates and Illumina sequencing libraries prepared using the KAPA Low-Throughput Library Preparation Kit with Standard PCR Amplification Module as previously described (Parker et al., 2015). The libraries were sequenced on an Illumina MiSeq instrument using the MiSeq reagent kit v2 (500-cycles; Illumina) following the manufacturer’s protocols. Illumina PE reads for each strain were mapped to the appropriate reference *C. jejuni* genome [*C. jejuni* 81–176 chromosome sequence: (NC_008787)] using Geneious software (v10.2.3; Biomatters, Ltd., Auckland, New Zealand; Kearse et al., 2012) using the parameters of at least 20x coverage and 80% of reads representing the mutation. All identified mutations were confirmed by repeating the analysis using the breseq software (v0.24rc6) package (Deatherage and Barrick, 2014), and later through manual examination. The complete genome sequences of *C. jejuni* isolates were submitted to GenBank under the project PRJNA634604 (accession: SRR22024544–SRR22024548; SRR22024599–SRR22024608).

### Generation of *C. jejuni* mutants and complementation constructs

All modified strains were created through homologous recombination using suicide vectors constructed using In-fusion cloning (Takara Bio Inc., Mountain View, CA) and/or standard restriction enzyme-mediated cloning to link the desired DNA fragments. The suicide vectors were generated by the amplification of DNA flanking regions and combined with a PCR-amplified chloramphenicol resistance cassette (SacII) and the pBSK-Kan2 vector backbone (Talukdar et al., 2020). The orientation of the DNA gene flanking fragments was XhoI/SacII upstream and SacII/SacI downstream. The specific suicide vector constructs were electroporated into *C. jejuni* to generate the desired isolates through homologous recombination. Electroporations were performed using a Bio-Rad *E. coli* Pulser and 2 mm gap cuvettes at the 2.5 kV voltage setting. *C. jejuni* mutants (∆*eptC*, ∆*mutY*, and ∆*uvrC*) were selected on MHB agar supplemented with 8 μg/ml chloramphenicol. Constructs were verified by restriction digestion and sequencing.

The plasmid vector prRNAHygR was used to construct the complement plasmids (Gourley et al., 2017). Briefly, a fragment of DNA harboring the gene of interest and upstream native promoter region was PCR amplified and cloned into the prRNAHygR vector using XbaI-BamHI sites. The complementation plasmids were electroporated into the *C. jejuni* mutants, and the transformants were selected on MHB agar plates supplemented with 250 μg/ml hygromycin. Constructs were verified by restriction digestion and PCR using gene-specific primers. A list of the specific primers and constructs is presented in Supplementary Table S1.

### EptC phenotypic assays

Motility assays of *C. jejuni* isolates were performed as previously described elsewhere (Malik-Kale et al., 2007; Neal-McKinney and Konkel, 2012). Briefly, overnight grown *C. jejuni* culture was suspended in MH broth at an OD_540_ of 0.1 and 3 μl of the bacterial suspension was spotted on an MH soft-agar plate (0.4% agar) and incubated for 48 h in microaerophilic condition. The image of the soft agar plate was captured with a GE ImageQuant LAS-4000 mini and the zone of motility was measured. The antimicrobial susceptibility test with polymyxin B was performed using the broth microdilution technique as described above. To determine the sensitivity of the isolates to DOC, isolates were grown in MH broth and MH broth supplemented with 0.05% Na-DOC for 24 h. Following incubation, the OD_540_ values of each culture were measured in triplicate.

### Statistical analysis

All assays were performed in triplicate to ensure reproducibility. Statistical analysis was performed using GraphPad Prism (v9.3.1).

## Results

### I. Exposure to DOC induces point mutations and ciprofloxacin resistance in *C. jejuni*

We found that growing *C. jejuni* in conditions to mimic the host-gut environment (medium supplemented with 0.05% Na-DOC) resulted in the production of intracellular ROS, which in turn caused DNA damage (Negretti et al., 2017). DNA damage was evident from the increased generation of the mutagenic 8-oxo-dG lesion and DNA breaks. While efficient and accurate repair of DNA damage is crucial to ensure genome stability and cell survival, we hypothesize that culturing *C. jejuni* with 0.05% DOC increases point mutations in the genome. To test this hypothesis, we examined the growth of *C. jejuni* in MH, a nutrient-rich broth, and MH supplemented with 0.05% Na-DOC. Ciprofloxacin resistance was chosen as a marker for DOC-induced mutations, focusing on the established mechanism of GyrA mutations at residue 86 or 90.

### IA. *C. jejuni* growth in DOC and selection of ciprofloxacin-resistant isolates

The serial passage of *C. jejuni* in MH and MH-DOC supplemented broths for 10 days resulted in ciprofloxacin-resistant colonies for both conditions. A total of 150 ciprofloxacin-resistant individual colonies (75 ciprofloxacin-resistant *C. jejuni* isolates grown with Na-DOC and 75 isolates grown without Na-DOC) were picked at random. A fragment of the *gyrA* gene was PCR-amplified for each of the isolates, using gene-specific primers, for sequencing to determine if a change had occurred in the QRDR at residue 86 or 90 (Supplementary Figure S1). Two isolates with point mutations in position 86 [ATA_86_, ACA (Thr) to ATA (Ile) and AAA_86_, ACA (Thr) to AAA (Lys)] and two in position 90 [TAT_90_, GAT (Asp) to TAT (Tyr) and AAT_90_, GAT (Asp) to AAT (Asn)] were selected among the isolates grown in either MH or MH-DOC broths. These point mutations in the *gyrA* gene have been previously reported in *C. jejuni* (Changkwanyeun et al., 2016). We observed a higher proportion of isolates with the GAT (Asp) to TAT (Tyr) signature at position 90 when the bacteria were co-cultured with DOC versus bacteria cultured in MH alone in at least two different runs. The increase in recovery of ciprofloxacin-resistant isolates with the G ➔ T transversion suggests that growth in DOC results in the production of intracellular ROS and an increase in the mutagenic 8-oxo-dG lesion.

### IB. GyrA variants confer different levels of ciprofloxacin resistance but similar growth rates

**Figure 1:**
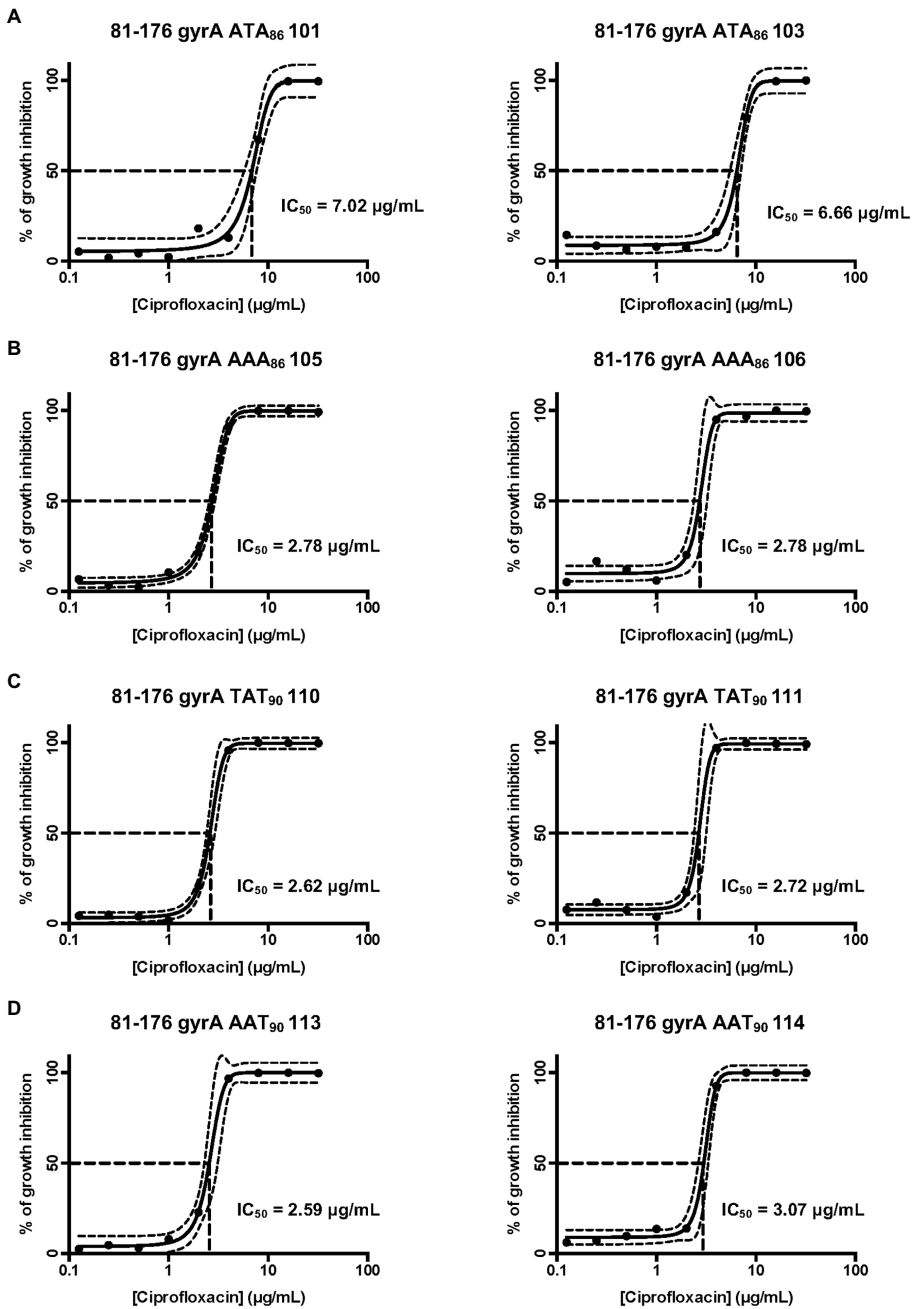
Concentration-effect curves for ciprofloxacin on select *C. jejuni* 81–176 isolates. **(A)** Isolates containing the ACA (Thr) to ATA (Ile) mutation at residue 86; **(B)** Isolates containing the ACA (Thr) to AAA (Lys) mutation at residue 86; **(C)** Isolates containing the GAT (Asp) to TAT (Tyr) mutation at residue 90; and **(D)** Isolates containing the GAT (Asp) to AAT (Asn) at residue 90. The antimicrobial activity of ciprofloxacin was determined by the broth microdilution method, as described in the section “Materials and methods”. Bacterial cultures were treated with different concentrations of ciprofloxacin and incubated for 48 h in a microaerobic chamber. The data represent a minimum of three biological replicates. The IC_50_ (50% of inactivated cells) was determined for each isolate. The dashed lines at right angles indicate the ciprofloxacin concentration that is required for a 50% reduction of the cells (IC_50_). The lines on both sides of the concentration-effect curve represent the 95% confidence intervals.

Since the DOC induces point mutations in the *gyrA* gene and thus confers ciprofloxacin resistance, we wanted to quantify the effect of each of the point mutations in the *gyrA* gene on ciprofloxacin resistance and bacterial growth. Concentration-effect curves for ciprofloxacin were generated for the *C. jejuni* ciprofloxacin-resistant isolates and the IC_50_ (50% of inactivated cells) was determined for each of the isolates. Reported are the IC_50_ values for two ATA_86_ isolates, two AAA_86_ isolates, two TAT_90_ isolates, and two AAT_90_ isolates. The IC_50_ was determined for two isolates with each mutation to reduce the chances of selecting an isolate with a second mutation that had occurred elsewhere in the genome that could provide enhanced ciprofloxacin resistance. The IC_50_ values of two ATA_86_ isolates were 7.0 μg/ml and 6.7 μg/ml, which were greater than the other isolates (AAA_86_, TAT_90_, and AAT_90_; Figure 1A). The other isolates with the three signature mutations had similar IC_50_ values; the IC_50_ of AAA_86_ isolates was 2.8 μg/ml (Figure 1B), the IC_50_ of TAT_90_ isolates were 2.6 μg/ml and 2.7 μg/ml (Figure 1C), and the IC_50_ of AAT_90_ isolates were 2.6 μg/ml and 3.1 μg/ml (Figure 1D). Next, the growth of all *C. jejuni* ciprofloxacin-resistant isolates was determined to see whether the differences in ciprofloxacin resistance alter bacterial growth in MH broth. Regardless of the inherent differences in ciprofloxacin resistance, the ciprofloxacin-resistant isolates demonstrated similar growth rates to each other and the wild-type strain in MH broth (Supplementary Figure S2).

### IC. Exposure to DOC increases *C. jejuni* ciprofloxacin-resistance mutation frequency and spectra

**Figure 2:**
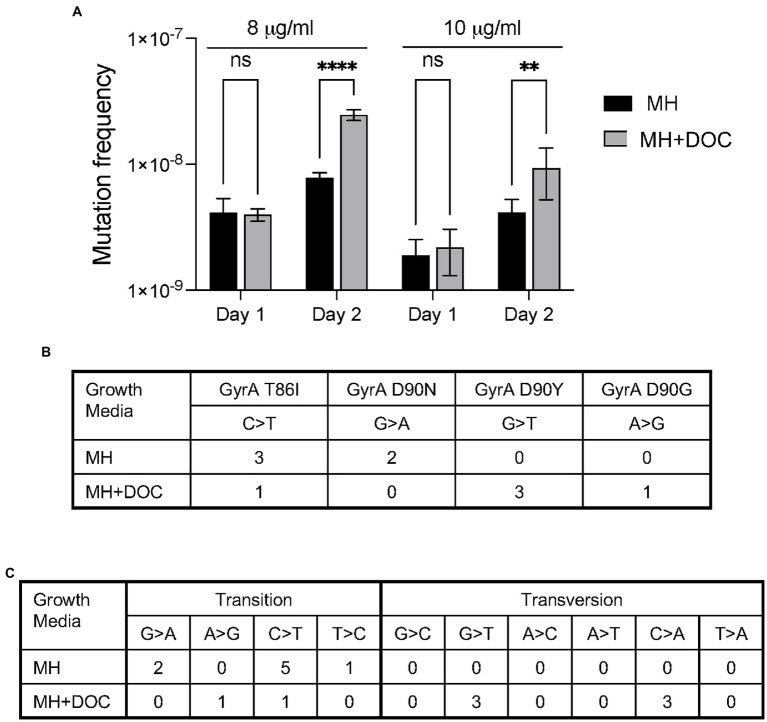
Culturing *C. jejuni* with deoxycholate (DOC) increases ciprofloxacin mutation frequency and causes an increase in DNA transversions. **(A)** *C. jejuni* were grown in MH and MH supplemented with sodium deoxycholate (Na-DOC) as outlined in “Materials and methods” and spread onto MHB plates without ciprofloxacin, MHB plates with 8 μg/ml of ciprofloxacin, and MHB plates with 10 μg/ml of ciprofloxacin. The resultant colonies were counted. The mutation frequency was calculated by dividing the CFU of *C. jejuni* ciprofloxacin-resistant by the total CFU. The data on Day 1 indicates the number of bacteria recovered from the bacteria grown in MH broth for one day and in MH broth supplemented with 0.05% Na-DOC for one day. The data are from a single experiment that was representative of three independent assays. Statistical analysis performed: Two-way ANOVA with Sidak’s multiple comparison test (**p < 0.01; ****p < 0.001, ns, non-significant). **(B,C)** Isolates were recovered after a serial passage in MH and MH supplemented with Na-DOC for 10 days as outlined in “Materials and Methods,” and five colonies from each culture were subjected to whole genome sequencing. Culturing *C. jejuni* in MH-DOC supplemented broth versus MH broth resulted in a higher proportion of isolates with DNA transversions at residue 90 of the *gyrA* gene **(B)**. Similarly, WGS revealed more DNA transversions in the isolates recovered from the MH-DOC versus the MH broth **(C)**.

In this study, we used the *gyrA* gene and ciprofloxacin resistance to determine the mutation frequency of *C. jejuni* grown in the presence and absence of DOC, as a single point mutation (transition and/or transversion) confers ciprofloxacin resistance. Thus, ciprofloxacin resistance is a proxy for genome-wide mutations. To determine if DOC increases the mutation frequency of the *gyrA* gene, *C. jejuni* was grown in a flask containing MH broth and in a flask with MH broth supplemented with 0.05% Na-DOC. Following a 24 h incubation period, the cultures were processed, and suspensions were spread onto MHB agar plates and MHB agar plates supplemented with 8 μg/ml and 10 μg/ml of ciprofloxacin. The preliminary assays with MHB agar supplemented with different concentrations (2, 4, 8, 10, and 12 μg/ml) of ciprofloxacin showed that only ATA_86_ isolates grew well on plates containing 10 μg/ml of ciprofloxacin. The other ciprofloxacin-resistant isolates either did not grow or grew poorly on plates containing 10 μg/ml of ciprofloxacin. However, all ciprofloxacin-resistant isolates, including the TAT_90_ isolates grew well on plates containing 8 μg/ml of ciprofloxacin. The ciprofloxacin mutation frequency for the bacteria grown in MH did not significantly change throughout the experiment. In addition, a significant difference was not observed in the mutation frequencies of the bacteria grown in the MH-DOC medium when compared to the MH medium for 1 day when plated on 8 or 10 μg/ml of ciprofloxacin (Figure 2A). However, an increase in the number of *C. jejuni* ciprofloxacin-resistant isolates was observed for the MH-DOC sample plated on 8 and 10 μg/ml of ciprofloxacin versus the MH culture on day 2; a three-fold increase was observed in the frequency of the ciprofloxacin-resistant isolates at 8 μg/ml (*p* < 0.001), and a two-fold increase was observed at 10 μg/ml (*p* < 0.01; Figure 2A). The increase in the number of colonies recovered on 8 μg/ml versus 10 μg/ml of ciprofloxacin, representing an increase in mutation frequencies, likely represented the recovery of isolates with point mutations that do not confer a high level of ciprofloxacin resistance. The increase in mutation frequency observed from day 1 to day 2 with DOC coincided with an increase in bacteria growth in the flask (not shown). Little change was observed in the ciprofloxacin-mutation frequency for the bacteria grown in DOC from day 3 to day 5 (not shown). In summary, *C. jejuni* growth in DOC increased in *gyrA* mutation frequency.

Five *C. jejuni* isolates grown in MH broth and five isolates grown in 0.1% Na-DOC broth for 10 days were selected for whole genome sequencing (WGS) to observe the mutation spectra (Figures 2B,C). In contrast to *C. jejuni* growth in MH, growth in Na-DOC resulted in an increase in DNA transversions versus transitions. The difference in mutation spectra was evident in *gyrA* as well as elsewhere in the genome. A transition is where a purine nucleotide is changed to another purine (A ↔ G), or a pyrimidine nucleotide is changed to another pyrimidine (C ↔ T). A transversion is a point mutation in which a purine (A or G) is changed to a pyrimidine (T or C, one ring) or vice versa. In essence, transitions involve bases of similar shapes, whereas transversions involve bases of different shapes (one ring versus two rings). The types of mutations (G ➔ C and C ➔ A transversions) identified in the genomes of the *C. jejuni* grown in Na-DOC are consistent with the published data indicating that DOC results in increased levels of ROS and 8-oxo-dG lesions. In summary, *C. jejuni* growth in DOC resulted in a change in the mutation spectra.

### II. Exposure to DOC induces mutations that lead to increased fitness/adaptation to DOC

We hypothesize that the increased frequency of the ciprofloxacin-resistant isolates at 8 μg/ml versus 10 μg/ml is primarily due to an increase in TAT_90_ isolates, resulting from the G ➔ T transversion, as preliminary assays revealed differences in the AAT_90_ isolates over the course of a 10-day passage in DOC-supplemented media. We further hypothesized that the increased recovery of ciprofloxacin-resistant isolates with G ➔ T transversions in the presence of DOC occurs because growth in this medium causes an increase of 8-oxo-dG lesions in the DNA. Based on this hypothesis and our preliminary findings, assays were performed to determine if *C. jejuni* growth in DOC could result in mutation(s) providing a fitness advantage or increased adaptation to DOC.

### II. Adaptation of *C. jejuni* to growth in medium with DOC

**Figure 3:**
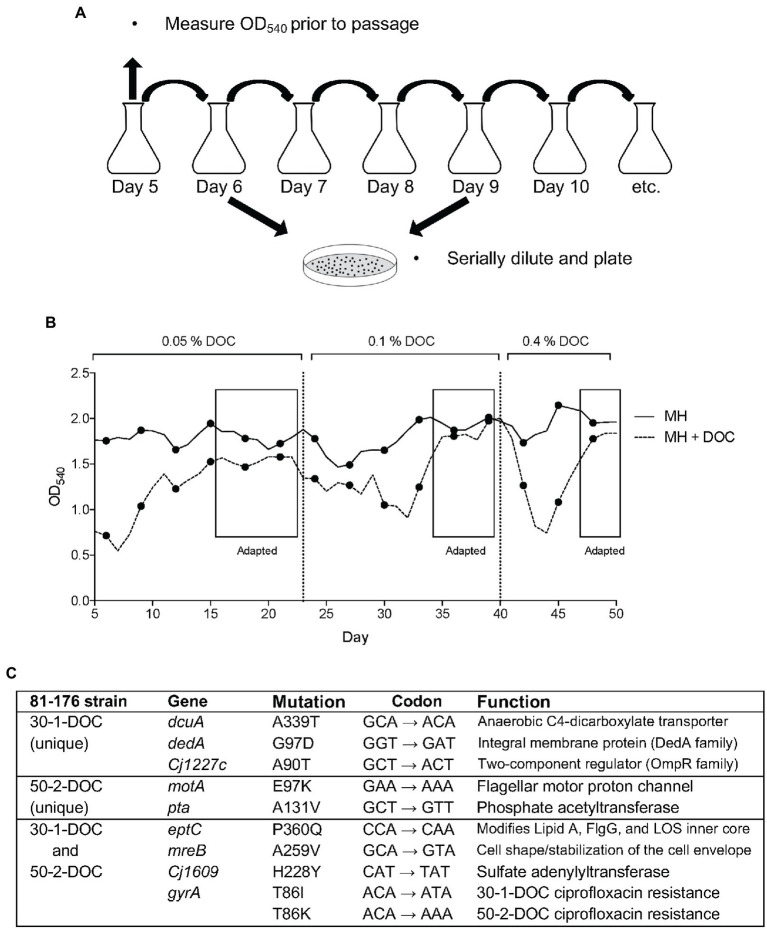
Adaptation of *C. jejuni* to growth in medium with deoxycholate (DOC). **(A)** Method of serial passage used to recover *C. jejuni*-DOC adapted isolates. Serially diluted aliquots of the bacterial growth cultures were spread onto MHB agar plates containing ciprofloxacin. **(B)** The optical density (OD_540_) values were generated from serially passaged cultures (samples were diluted to be in the linear range of the instrument). **(C)** Genomic mutations in two *C. jejuni* isolates 30-1-DOC and 50-2-DOC grown in a medium with DOC recovered after 30 and 50 days of passages, respectively.

To determine a specific genomic mutation that occurs upon *C. jejuni* passage in DOC, an experiment was performed whereby the bacteria were subjected to repeated passage in media containing a stepwise increase in the concentration of Na-DOC (from 0.05% ➔ 0.1% ➔ 0.4%; Figure 3A). A slightly lower bacterial concentration (OD_540_ of 0.04) was used to inoculate the cultures after each day of growth for this experiment than for the ciprofloxacin mutation frequency experiment (described in Results section IC), as we thought this strategy would result in greater pressure for the bacteria to adapt. For the DOC-supplemented culture, the bacteria were grown in MH broth with 0.05% Na-DOC and passaged every day until the OD_540_ for the Na-DOC-supplemented culture was similar to the MH culture over a several-day period (days 16–22; Figure 3B). On day 23, the concentration of Na-DOC in the medium was increased to 0.1%, and the bacterial culture was passaged in 0.1% Na-DOC-supplemented media until the OD_540_ value had increased and was ‘similar’ to that of the bacteria cultured in MH medium alone at day 34–39 (Figure 3B). At day 40, the concentration of Na-DOC in the medium was increased from 0.1% ➔ 0.4%, and the bacterial culture was passaged in 0.4% Na-DOC-supplemented media up to day 50. On days 47–50, the OD_540_ of the MH-DOC culture was similar to the MH culture. Several isolates were picked every few days during the experiment and were frozen. At the conclusion of the experiment, the resistance of selected isolates to DOC was evaluated by measuring the growth (OD_540_) of the bacteria after a 24-h incubation period (Supplementary Figure S3).

Two representative *C. jejuni* isolates (30-1-DOC and 50-2-DOC) that demonstrated increased resistance to Na-DOC after 30 and 50 days of serial passages were selected for WGS, and the data was compared to the sequence of the wild-type parental strain. Illumina sequencing revealed mutations in seven genes in the *C. jejuni* 30-1-DOC isolate and six genes in the *C. jejuni* 50-2-DOC isolate compared to the wild-type isolate (Figure 3C). The *C. jejuni* 30-1-DOC and 50-2-DOC isolates both contained a C ➔ A mutation in the *eptC* gene, indicative of a G ➔ T transversion on the non-coding strand. Given that both *C. jejuni* 30-1-DOC and 50-2-DOC contained a G ➔ T transversion at residue 60 [CCA (Pro) to CAA (Gln), P360Q mutation] in the *eptC* gene, additional studies were performed to determine if a modification of the EptC native protein would alter an isolate’s phenotypic properties. The *eptC* gene encodes for a phosphoethanolamine (pEtN) transferase that modifies a flagellar rod protein, a domain on the lipooligosaccharide (LOS), and several N-linked glycans (Cullen et al., 2013). The *eptC* gene is 1,784 bp in length and encodes a protein of 60 kDa (Supplementary Figure S4).

### IIB. Phenotypic characterization of a *C. jejuni eptC* mutant

To explore the relationship between DOC and an *eptC* mutation, an *eptC* deletion mutant (Δ*eptC*) was generated and the mutant was complemented with a copy of the wild-type gene or a copy of the gene containing the P360Q mutation. The *C. jejuni* wild-type strain and EptC variants were tested for motility, sensitivity to polymyxin B, and resistance to DOC. Others have reported that a *C. jejuni* Δ*eptC* has reduced motility and increased polymyxin B sensitivity compared to a wild-type isolate (Scott et al., 2012; Fage et al., 2014; Lim and Kim, 2017).

Motility assays revealed that the Δ*eptC* mutant was impaired in motility when compared to the wild-type strain, which is in agreement with previous reports (Cullen et al., 2012; Scott et al., 2012; Fage et al., 2014). Transformation of the Δ*eptC* mutant with a wild-type copy of the *eptC* gene (EptC_WT_) and the *eptC* gene harboring the CCA to CAA point mutation (P360Q variant, EptC_P360Q_) restored the motility of the isolates to nearly that of the wild-type strain (Supplementary Figure S5).

**Figure 4:**
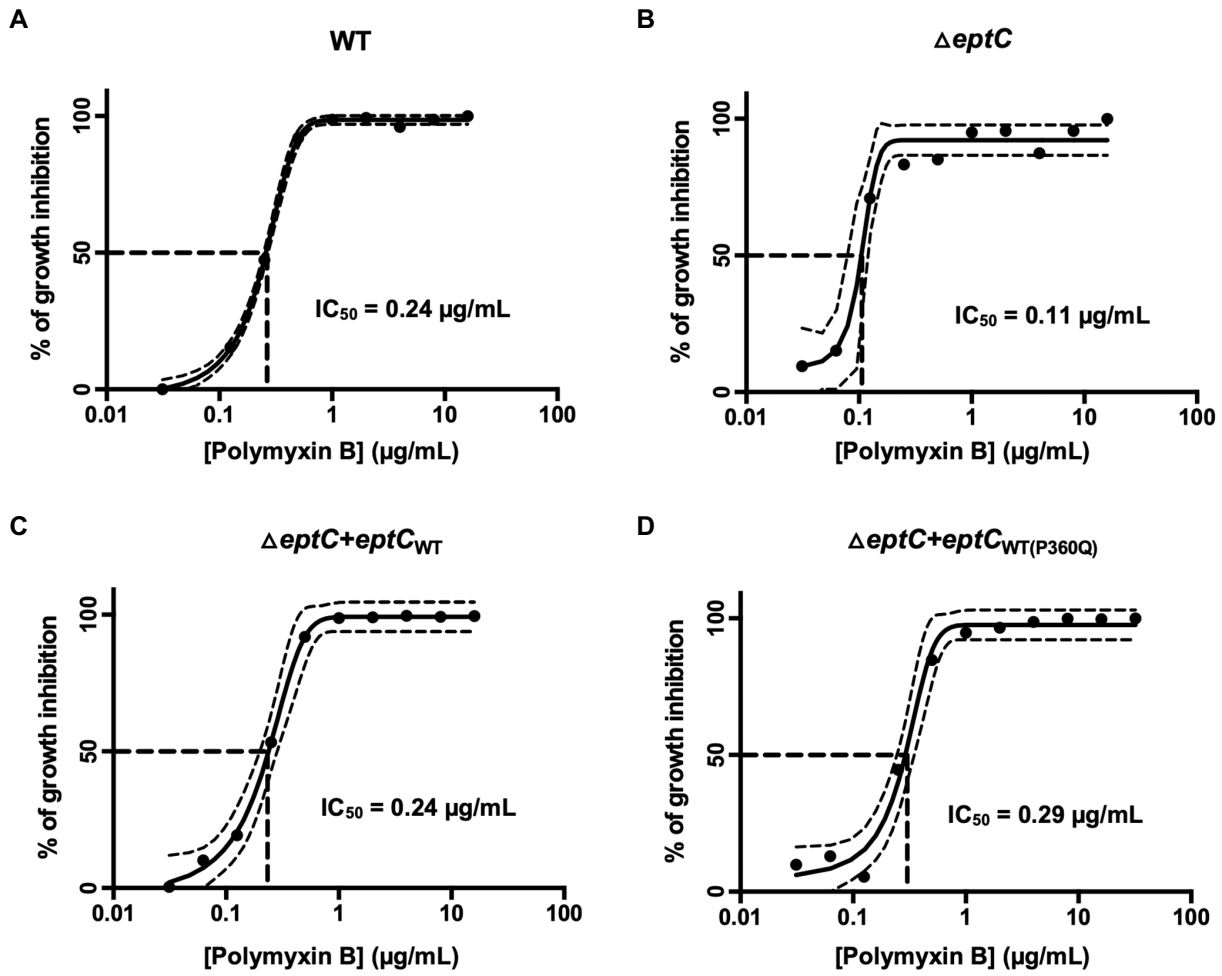
A *C. jejuni* △*eptC* mutant shows enhanced sensitivity to polymyxin B, whereas a *C. jejuni* variant synthesizing the EptC P360Q protein demonstrates enhanced resistance to polymyxin B. Concentration-effect curves for polymyxin B against a *C. jejuni* **(A)** wild-type strain, **(B)** △*eptC* deletion mutant, **(C)** △*eptC* deletion mutant transformed with a gene expressing the EptC_WT_ protein (△*eptC* + *eptC*_WT_), and **(D)** △*eptC* deletion mutant transformed with a gene expressing the EptC protein containing the P360Q variant (△*eptC* + *eptC*_WT(P360Q)_). The antimicrobial activity of polymyxin B was determined by the broth microdilution method, as described in the section “Materials and methods”. The data represent a minimum of three biological replicates. Dashed lines at right angles indicate the polymyxin B concentration that is required for a 50% reduction of the cells (IC_50_). The lines on both sides of the concentration-effect curve represent the 95% confidence intervals.

Polymyxin B is a positively charged lipopeptide that binds to the negatively charged surface of Gram-negative bacteria and disrupts the outer cell membrane. The Δ*eptC* mutant demonstrated increased sensitivity to polymyxin B when compared to the wild-type strain (Figure 4), which is again consistent with previous studies (Cullen et al., 2012; Scott et al., 2012; Fage et al., 2014). Synthesis of the EptC_WT_ protein in the Δ*eptC* mutant demonstrated a level of polymyxin B resistance nearly to the same level as the *C. jejuni* wild-type strain. However, unexpectedly, synthesis of the EptC_P360Q_ protein in the Δ*eptC* mutant demonstrated polymyxin B resistance at a level greater than observed for the wild-type strain (Figure 4).

**Figure 5:**
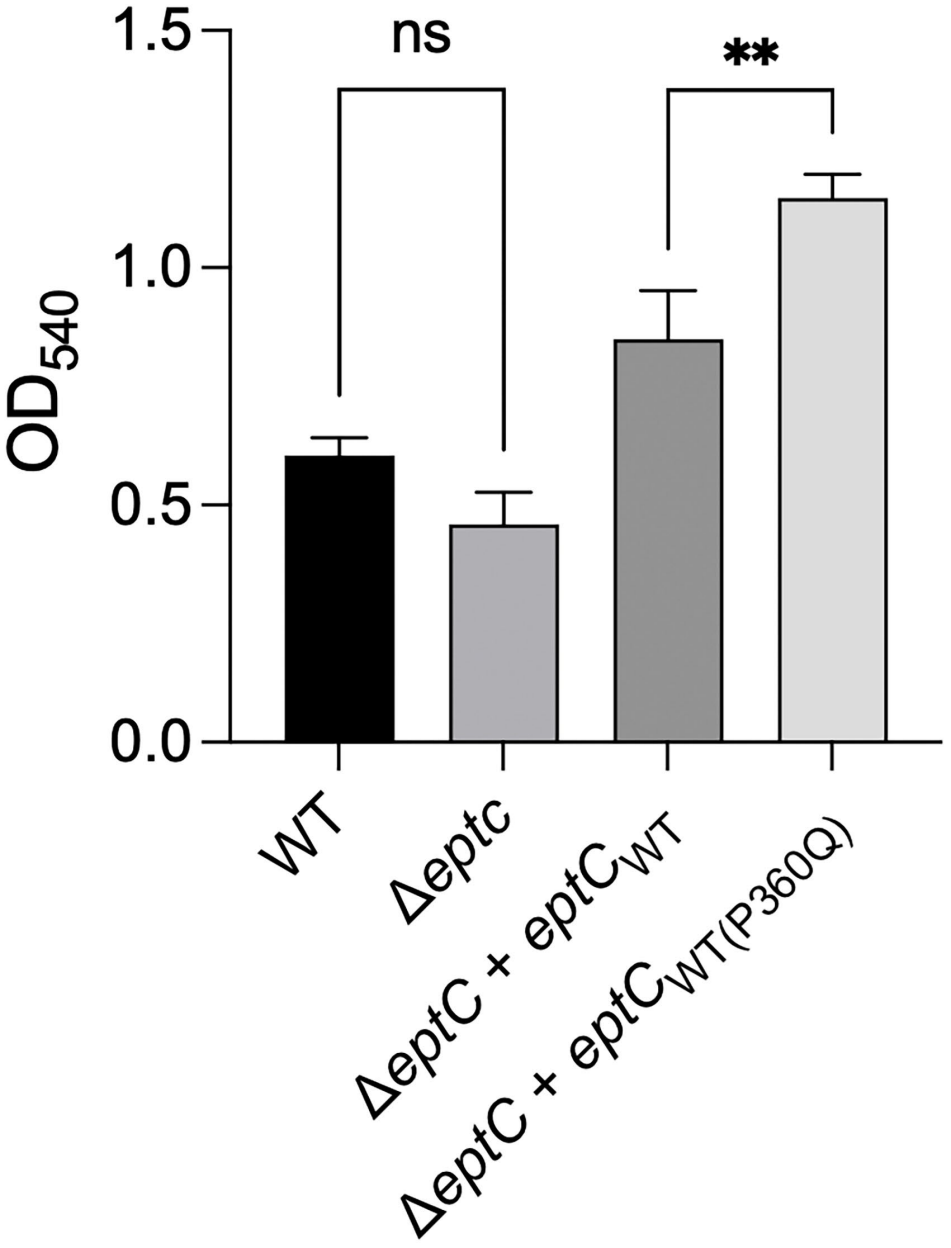
A *C. jejuni* variant synthesizing the EptC P360Q protein demonstrates enhanced growth in deoxycholate (DOC)-supplemented medium. A *C. jejuni* wild-type strain, △*eptC* deletion mutant, △*eptC* deletion mutant transformed with a gene expressing the EptC_WT_ protein (△*eptC* + *eptC*_WT_), and △*eptC* deletion mutant transformed with a gene expressing the EptC protein containing the P360Q variant (△*eptC* + *eptC*_WT(P360Q)_) were grown in a microaerobic chamber at 37°C with shaking for 24 h in MH medium supplemented with 0.05% Na-DOC. The data represent the mean ± standard deviation of the terminal OD_540_. Statistical analysis performed: One Way ANOVA with Tukey’s multiple comparison test (**p < 0.01, ns, non-significant).

**Figure 6:**
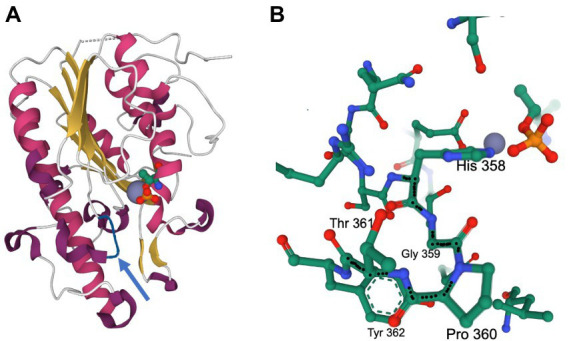
Mutation of the Pro to Gln at residue 360 alters the activity of the *C. jejuni* EptC enzyme. Pro-360-Gln mutations were recovered after culturing with sodium deoxycholate. The structure of *C. jejuni* EptC highlights the His_358_-Gly_359_-Pro_360_ sequence adjacent to the active site. The figures were generated using the pdb file 4TN0. **(A)** Ribbon diagram of the C-terminal periplasmic domain of EptC. The location of the active site is indicated by the Zn^2+^ ion (gray sphere) and the catalytically important Thr_266_, which is phosphorylated, mimicking a reaction intermediate (CPK representation, with phosphate in yellow and red). The loop containing the His-Gly-Pro motif is shown in blue. **(B)** Environment within 5 Å of the His-Gly-Pro motif. This rendering demonstrates the proximity of this motif to the active site groups (phospho-Thr and Zn^2+^). The structure also highlights the tight turn achieved by the Gly_359_-Pro_360_ sequence preceding the helix initiated by Thr_361_–Tyr_362_ (also shown); the backbone of the His_358_-Gly_359_-Pro_360_-Thr_361_ sequence is highlighted by black dots.

The growth of the Δ*eptC* mutant and Δ*eptC* mutant synthesizing either the EptC_WT_ protein or EptC_P360Q_ variant protein was then examined in a medium supplemented with 0.05% Na-DOC. No significant difference was observed in the growth of the Δ*eptC* mutant in the DOC-supplemented medium when compared to the wild-type strain (Figure 5). However, an increase was observed in the growth of the Δ*eptC* transformant synthesizing the EptC_WT_ protein when compared to the wild-type isolate. More interestingly, a significant increase was observed in the growth of the Δ*eptC* isolate synthesizing the EptC_P360Q_ protein versus the Δ*eptC* transformant expressing the EptC_WT_ protein. We speculate that the replacement of the Pro, an amino acid that disrupts structures with a hydrophilic residue (Gln) at position 360 of EptC (Figure 6), alters which acceptor molecules that can be modified with pEtN, leading to increased resistance to DOC and polymyxin B. Together, these data indicate that the C ➔ A mutation, resulting in a P360Q codon change, enhances the fitness of an isolate to a cationic antimicrobial peptide (polymyxin B) and a secondary bile salt (DOC).

### III. MutY repairs G to T mutations caused by DOC

*C. jejuni* possesses DNA repair systems that function in BER, NER, and HRR but lacks a functional mismatch repair system (Table 1). *C. jejuni* growth in DOC results in increased levels of ROS and 8-oxo-dG. If an 8-oxoG: a base pair is generated during replication, a DNA glycosylase termed MutY excises the adenine to avoid making the mutation permanent in the next round of replication (Banda et al., 2017). We hypothesized that a deletion of the *mutY* gene would increase global genome mutations because non-bulky lesions (repaired by BER) can be bypassed by DNA polymerases. If the hypothesis is correct, we expect to observe an increase in the frequency of ciprofloxacin resistance.

**Table 1:** List of *C. jejuni* genes that share similarities to DNA repair systems in *Escherichia coli*.

DNA repair system	*C. jejuni* gene (NCTC 11168^1^/81–176^2^)	*E. coli* gene (K12^3^)	In operon? (Yes/No; position in operon)	% Identity/% Similarity^4^
*Homologous recombination*				
RecA	Cj1673c/CJJ81176_RS08060	b2699	Yes (1/2)	57.8%/76.0%
RecB*	Cj1481/CJJ81176_RS07100	b2820	Yes (2/2)	17.1% /32.7%
RecC*	Cj1482/CJJ81176_RS07105	b2822	Yes (1/2)	12.4%/24.8%
RecD*	n/a	b2819	n/a	n/a
RecF	n/a	b3700	n/a	n/a
RecJ	Cj0028/CJJ81176_RS00140	b2892	No	28.3%/49.2%
RecN	Cj0642/CJJ81176_RS03125	b2616	Yes (2/2)	21.8%/42.2%
RecO	Cj0120/CJJ81176_RS00635	b2565	Yes (19/20)?	13.1%/23.9%
RecQ	n/a	b3822	n/a	n/a
RecR	Cj1263/CJJ81176_RS06150	b0472	Yes (3/3)	31.2%/54.1%
SSBP	Cj1071/CJJ81176_RS05200	b4059	Yes (2/3)	33.0%/47.6%
*Nucleotide excision repair*				
UvrA	Cj0342c /CJJ81176_RS01645	b4058	Yes (1/2)	50.5%/67.6%
UvrB	Cj0680c /CJJ81176_RS03285	b0779	No	50.1%/69.2%
UvrC*	Cj1246c /CJJ81176_RS06065	b1913	Yes (2/3)	29.1%/49.1%
UvrD	Cj1101/CJJ81176_RS05350	b3813	Yes (5/13)	34.5%/53.3%
Mfd	Cj1085c /CJJ81176_RS05270	b1114	Yes (10/15)?	28.2%/46.6%
*Base excision repair*				
MutY*	Cj1620c /CJJ81176_RS07755	b2961	No	31.8%/50.4%
MutM	n/a	b3635	n/a	n/a
Nei	n/a	b0714	n/a	n/a
Nth	Cj0595c /CJJ81176_RS02900	b1633	Yes (1/3)	38.6%/56.7%
Tag	n/a	b3549	n/a	n/a
AlkA	n/a	b2068	n/a	n/a
Ung	Cj0086c /CJJ81176_RS00465	b2580	No	43.8%/61.7%
XthA/ExoA*	Cj0255c /CJJ81176_RS01235	b1749	No	32.4%/49.2%
Nfo	n/a	b2159	n/a	n/a
Udg	Cj0963/CJJ81176_RS04690		Yes (1/3)	
*Mismatch repair*				
MutS	Cj1052c /CJJ81176_RS05110	b2733	Yes (2/12)	11.3%/22.0%
MutL	n/a	b4170	n/a	n/a
MutH	n/a	b2831	n/a	n/a
*Direct repair*				
Ada	n/a	b2213	n/a	n/a
AlkB	n/a	b2212	n/a	n/a
Ogt	n/a	b1335	n/a	n/a
Phr	n/a	b0708	n/a	n/a

n/a, Not applicable.

1 *C. jejuni* NCTC 11168 GenBank accession number: NC_002163.1.

2 *C. jejuni* 81–176 GenBank accession number: NC_008787.

3 *E. coli* K-12 GenBank accession number: AP009048.

4 Identity and similarity between *C. jejuni* 81–176 and *E. coli* K-12.

* Genes targeted for mutagenesis (gene deletion).

### IIIA. A *mutY* deletion results in a hyper-mutation phenotype yielding increased ciprofloxacin resistance

To test the hypothesis that the BER system in *C. jejuni* is involved in counteracting ROS-induced stress, the three DNA repair systems were targeted by functionally disrupting each pathway. Gene deletions were generated in *addAB* (HRR), *uvrC* (NER), and *mutY* (BER). The growth and the mutation frequency of the Δ*uvrC* and Δ*addAB* mutants were observed in MH and MH-DOC supplemented media. The mutants (Δ*uvrC* and Δ*addAB*) were grown over a 36-h incubation period and the OD_540_ was measured. The *C. jejuni* wild-type, Δ*uvrC* and Δ*addAB* mutants showed impaired growth with MH-DOC compared to MH only (Supplementary Figures S6A–C). To determine the ciprofloxacin mutation frequency, the bacteria were grown in MH and MH-DOC supplemented media and passaged every 24 h for a 10-day incubation period. The mutation frequency decreases when either Δ*uvrC* or Δ*addAB* mutants are grown with MH-DOC compared to the MH only over 10 days (Supplementary Figures S6E,F), whereas the mutation frequency increased over time for wild-type isolates (Supplementary Figure S6D). Based on these findings, we focused our studies on analyzing the mutations in the *C. jejuni* wild-type strain and Δ*mutY* mutant in a medium with and without DOC.

**Figure 7:**
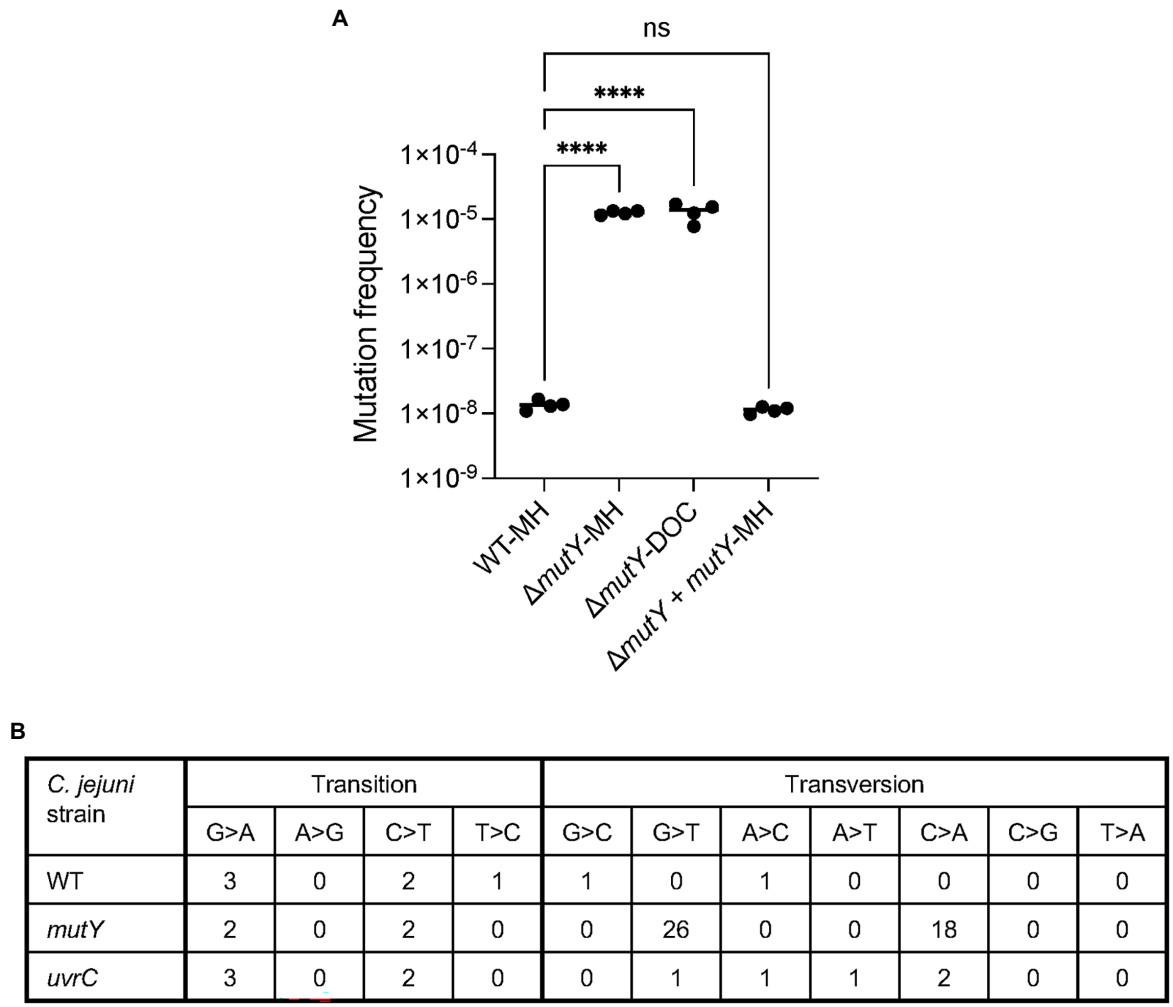
Mutation frequency and types of mutations demonstrated by the *C. jejuni* Δ*mutY* mutant. **(A)** The ciprofloxacin mutation frequency of a *C. jejuni* wild-type strain cultured in MH broth (WT-MH), Δ*mutY* mutant cultured in MH broth (Δ*mutY*-MH), and deoxycholate (DOC)-supplemented media (Δ*mutY*-DOC), and △*mutY* deletion mutant harboring a chromosomal copy of the *mutY* wild-type gene cultured in MH broth (Δ*mutY* + *mutY*-MH). *C. jejuni* were grown in MH and MH supplemented with 0.05% Na-DOC in a microaerobic incubator at 37°C with shaking for 24 h. Following incubation, the cultures were spread onto MHB plates and MHB plates with ciprofloxacin. The mutation frequency was calculated by dividing the CFU of *C. jejuni* ciprofloxacin-resistant by the total CFU. Statistical analysis performed: One Way ANOVA with Tukey’s multiple comparison test (****p < 0.001, ns, non-significant). **(B)** Types of mutations identified in the *C. jejuni* wild-type strain (WT), △*mutY* deletion mutant (Δ*mutY*), and △*uvrC* deletion mutant (Δ*uvrC*) mutant cultured in MH broth.

Growth of the Δ*mutY* mutant in MH broth resulted in a 500-fold increase or greater in ciprofloxacin mutation frequency than observed previously with the wild-type strain (*p* < 0.001; Figure 7A). Notable is that the ciprofloxacin mutation frequency observed for the Δ*mutY* mutant was similar in MH and MH-DOC supplemented media. However, when the Δ*mutY* mutant was transformed with the wild-type copy of the *mutY* gene, the ciprofloxacin mutation frequency was similar to the wild-type strain (not shown). Based on this finding and the data in Figure 2, we speculate that the growth of *C. jejuni* in DOC-supplemented medium results in levels of ROS that overwhelm the BER system. We concluded that BER plays a significant role in modulating *C. jejuni* mutation programs in response to DOC exposure.

### IIIB. A *mutY* deletion results in increased G to T and C to A transversions

The increased mutation frequency of the *C. jejuni mutY* deletion mutant prompted an investigation of the types of mutations that occurred in this genetic background versus that of the wild-type strain and *uvrC* deletion mutant. Point mutations [transitions, transversions, and indels (insertions and deletions)] were identified by culturing the bacteria in MH broth and performing WGS. The *C. jejuni* wild-type strain had no G ➔ T or C ➔ A transversions (Figure 7B). The *C. jejuni* Δ*uvrC* mutant had one G ➔ T and two C ➔ A transversions. In contrast, the *C. jejuni* Δ*mutY* had 26 G ➔ T and 18 C ➔ A transversions. Overall, the *C. jejuni* wild-type strain and Δ*uvrC* mutant had fewer transitions and transversions than the Δ*mutY* mutant. Next, we wanted to determine whether the serial passage of *C. jejuni* wild-type and Δ*mutY* mutants in MH had any effect on the number of G ➔ T and C ➔ A transversions. *C. jejuni* wild-type and Δ*mutY* mutant were grown in MH broth and passaged every 24 h for 10 days, and five individual colonies for each strain were randomly selected for sequencing. Consistent with the previous finding, there were a higher number of G ➔ T and C ➔ A transversions in the *C. jejuni* Δ*mutY* mutant compared to the wild-type strain grown in MH (not shown). These findings suggest that the absence of *mutY*, a.k.a. BER system in *C. jejuni*, increases the number of G ➔ T and C ➔ A transversions, ciprofloxacin resistance isolates, and, potentially, bacterial fitness.

## Discussion

**Figure 8:**
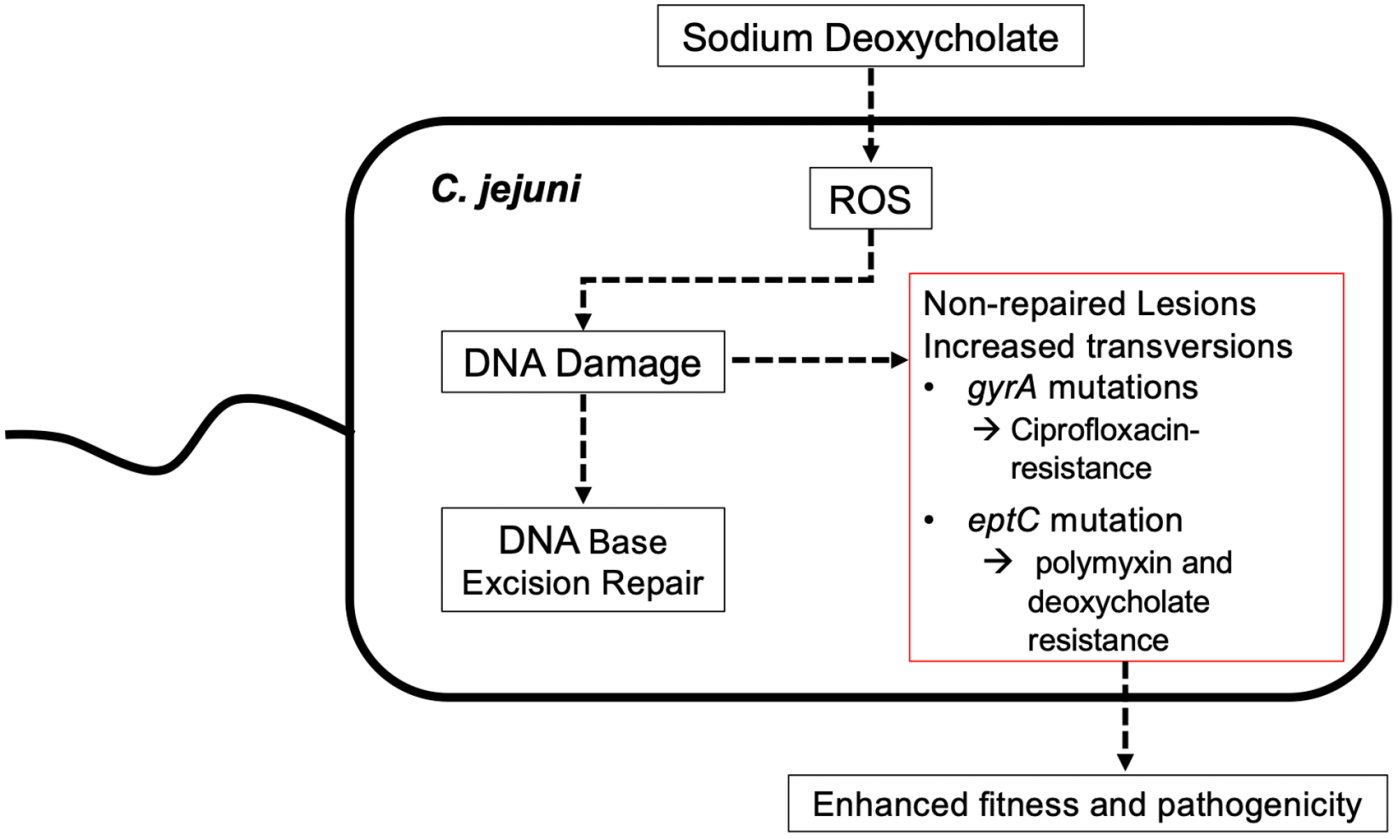
Model illustrating a potential pathway for how the growth of *C. jejuni* in a medium containing physiological concentrations of sodium deoxycholate drive bacterial mutations and adaptation. Previous work has demonstrated that the growth of *C*. *jejuni* in deoxycholate (DOC) results in increased levels of reactive oxygen species (ROS) and 8-oxo-dG:adenine base pairs. While likely that all DNA repair systems are necessary for repairing ROS-induced mutations, the base excision repair system is responsible for the repair of minor distortion in the DNA helix structure, including the 8-oxo-dG lesion that results from increased levels of ROS. The work presented here indicates that the growth of *C. jejuni* in DOC results in an increased mutation rate, including G to T transversions.

Genomic rearrangements have been observed in *C. jejuni* recovered from animals (Mixter et al., 2003; Barton et al., 2007; (Ridley et al., 2008). The generation of *C. jejuni* variants *in vivo* is of particular interest because this pathogen lacks the SOS machinery and mutagenic DNA polymerases that *E. coli* utilizes in response to stress. Within the gut of vertebrate hosts, *C. jejuni* is exposed to bile, a protective agent against bacterial colonization. We have shown that *C. jejuni* growth in a medium containing the bile salt DOC results in ROS production (Negretti et al., 2017). Guanine is the most susceptible of the four bases to oxidation, and its oxidation product, 8-oxo-dG, is a common lesion in DNA. We hypothesized that the *C. jejuni* DNA repair systems make errors, resulting in mutations (Figure 8). This study aimed to determine if host stressors, such as DOC, can induce genetic mutations and serve as a possible driver of pathogen evolution. We sought to determine if *C. jejuni* growth in a medium containing DOC alters the mutation frequency and has the potential to generate new clonal types with enhanced fitness.

In this study, we initially assessed the mutations in the *gyrA* gene, which confer ciprofloxacin resistance, to determine if *C. jejuni* growth in DOC alters the frequency/type of genomic mutations. The *gyrA* gene represents an ideal target for our study, as it is simple to assess ciprofloxacin resistance and because a variety of point mutations confer antibiotic resistance. We passaged *C. jejuni* in MH medium with and without DOC and readily recovered ciprofloxacin-resistant isolates with four mutations (Thr-86-Ile, Thr-86-Lys, Asp-90-Asn, and Asp-90-Tyr) in the QRDR of GyrA. As predicted from published reports, isolates with the GyrA Thr-86-Ile mutation demonstrated greater resistance to ciprofloxacin than the isolates with other point mutations, as judged by the IC_50_ values (Changkwanyeun et al., 2016). Although we selected the *C. jejuni* ciprofloxacin-resistant isolates cultured in media with and without DOC at random, only isolates with the Asp-90-Tyr (GAT to TAT) signature, a G ➔ T transversion was recovered in greater numbers when the bacteria were cultured with DOC. This result is consistent with the previous findings that *C. jejuni* growth in DOC-supplemented medium results in the production of intracellular ROS and an increase in the mutagenic 8-oxo-dG lesion, thus, a G ➔ T transversion in the DNA (Negretti et al., 2017). WGS of the *C. jejuni* grown in the presence of DOC revealed a greater number of DNA transversions versus transitions, as well as the Asp-90-Tyr (GAT to TAT) mutation, indicating that growth in DOC resulted in a change in the mutation spectra when compared to isolates grown in MH broth. Together, these data show that DOC increases the frequency of *C. jejuni* ciprofloxacin-resistant isolates versus background levels (i.e., *C. jejuni* grown in MH broth) in the absence of antibiotic pressure and shifts the mutations from transitions to transversions.

One question that arises from the apparent increase in *C. jejuni* ciprofloxacin-resistant isolates with the Asp-90-Tyr signature in our study is why these isolates are not more frequently recovered from poultry. We speculate that the lack of recovery of the Asp-90-Tyr signature in GyrA from poultry is due to the increased virulence potential and fitness of Thr-86-Ile isolates. Some *C. jejuni* strains with the Thr-86-Ile, but not with the Asp-90-Tyr signature, have relaxed DNA supercoiling activity (Changkwanyeun et al., 2016; Shortt et al., 2016). DNA gyrase acts by wrapping DNA into a positive supercoil, passing one DNA strand through the other by breakage, and then rejoining the DNA strand (Hawkey, 2003). FQs bind to the complexes between the DNA and gyrase enzyme, and prevent the re-ligation of the broken DNA strands. The residue at the position equivalent to Thr-86 is Ser or Thr in GyrA from several bacterial pathogens, with those containing Thr exhibiting greater resistance to ciprofloxacin (Bax et al., 2010; Blower et al., 2016). This residue forms a conserved hydrogen bond to the carboxylate of ciprofloxacin (Supplementary Figure S7). Replacement of Thr-86 with either Ile or Lys would result in loss of this interaction and additionally may cause steric clashes in the surrounding contacts with ciprofloxacin and the DNA termini. The equivalent residue to Asp-90 is conserved as Asp or Glu. It appears to contribute to the negative charges in the environment around the Mn^2+^ ion chelated by the carboxylate and hydroxyl groups on ciprofloxacin (Supplementary Figure S7). Similarly, a mutation of Asp-90 to either Tyr or Asn would remove the electrostatic interactions formed by Asp or Glu. Overall, the extent and conservation of interactions of the Thr-86 residue are significantly greater than those of the Asp-90 residue, consistent with the observation that mutation of Thr-86 causes greater resistance to ciprofloxacin, presumably due to weaker binding interactions with the protein: DNA complex. In addition, isolates with the Thr-86-Ile mutation demonstrate an increase in virulence phenotypes, including protein secretion and increased intestinal cell invasion (Scanlan et al., 2017; Whelan et al., 2019). *In vivo* experiments have revealed that a *C. jejuni* Thr-86-Ile isolate has enhanced fitness, as it can outcompete a wild-type isolate in poultry inoculated with a mixture of both isolates, i.e., the Thr-86-Ile isolate dominants and persist in the chickens (Luo et al., 2003). Finally, using the *in vivo Galleria mellonella* larvae model of infection, a Thr-86-Ile isolate was found to kill the larvae more quickly than a wild-type isolate (Whelan et al., 2019). It is possible that the low recovery of isolates with the Asp-90-Tyr signature from poultry is due to the low GC content of *Campylobacter* organisms. *C. jejuni* has mol% (G + C) content of ~30.4%, which might slightly decrease the frequency of guanine oxidation compared with enteric bacteria having mol% (G + C) content of ~50%. However, this does not explain the increased mutation frequency and G ➔ T transversion observed with the *mutY* mutant. Together, we propose that FQ-resistant isolates are prevalent in poultry, the natural reservoir, due to the enhanced fitness of the Thr-86-Ile isolates. We speculate that the increased residency of *C. jejuni* in the intestine of humans, as well as the increased bile concentration in the human intestine compared to poultry, provides an environment where the frequency and spectra of genomic mutations may be increased.

To test if DOC results in mutation(s) that provide enhanced bacterial fitness, *C. jejuni* was continually passaged in DOC-supplemented media and two isolates that demonstrated enhanced DOC resistance were subjected to WGS. Both isolates demonstrated mutations in several genes, including *eptC*. We focused on the *eptC* mutation in this study because a C ➔ A transversion was observed at position 360 (P360Q variant) and because the protein is involved in host-cell interactions. EptC catalyzes the addition of pEtN to various outer membrane components, including LOS and the flagellar rod protein FlgG. To determine whether the P360Q mutation in the EptC protein conferred a fitness advantage, we first generated an Δ*eptC* deletion mutant. In agreement with previous reports, the Δ*eptC* showed a defect in motility and increased sensitivity to polymyxin B (Cullen et al., 2013; Lim and Kim, 2017). Complementation of Δ*eptC* mutant, by the introduction of a wild-type copy gene driven by its endogenous promoter in the chromosome, restored the isolate’s motility and polymyxin B resistance to the level of the wild-type isolate.

Previous work with *Salmonella* spp. has revealed that bile resistance can be acquired through the modification of numerous factors, including lipopolysaccharide, proteins in the cytoplasmic and outer membranes, gene regulatory networks, and DNA repair systems (Hernandez et al., 2012). To determine if the EptC P360Q variant shows evidence of increased virulence potential, a gene encoding the EptC P360Q variant was introduced into the Δ*eptC* mutant. Noteworthy is that the EptC residue Pro-360 is central to a conserved sequence motif in this family of bacterial pEtN transferases that includes the essential active site residue His-358 implicated in metal ion binding and a constrained turn that includes the preceding Gly-359. The P360Q mutation does not eliminate EptC activity but appears to allow improved function, perhaps through broadening the substrate specificity of EptC.

A greater number of DNA transversions versus transitions were observed in the genomic sequences of the *C. jejuni* grown in a medium supplemented with DOC than without DOC. While one would predict that these mutations are repaired by BER pathway (Krokan and Bjørås, 2013), this has not formally been shown in *C. jejuni*. Thus, we analyzed the behavior and genomic background of a Δ*mutY* deletion mutant. In brief, we found an increase in G ➔ T and C ➔ A transversions in the genome of the *C. jejuni* Δ*mutY* isolate. In contrast, no transversions were observed in the genome of the wild-type isolate, and only a few transversions were observed in the Δ*uvrC* mutant. Relevant to this discussion is that the NER pathway serves as a secondary defense against oxidative damage and recombinational repair is also required under certain instances (e.g., single and double-strand breaks; Demple and Harrison, 1994). Finally, both the Δ*uvrC* mutant and Δ*addAB* mutants demonstrate decreased cell viability and mutation frequency when grown in a medium supplemented with DOC, possibly due to DNA lesions caused by oxidative damage. ROS can stall replicative polymerases during DNA replication, where 8-oxo-dG would allow mutagenic insertion and continued DNA synthesis. Based on these data, we concluded that the BER pathway plays the primary role in the repair of the 8-oxo-dG lesion in *C. jejuni*, but that all three DNA repair systems are involved in general oxidative damage repair.

We present data that demonstrate that the growth of *C. jejuni* in the bile salt DOC, causes DNA mutations that enhance the adaptation of *C. jejuni* to the conditions encountered in a host. While one gene product, EptC, was demonstrated to contribute to the enhanced resistance of polymyxin B (an antimicrobial agent) and DOC resistance, we postulate that the exposure of *C. jejuni* to DOC results in other mutations, which go un-repaired, that may result in either enhanced or reduced fitness of the organism over time. Discovering the single nucleotide polymorphisms (SNPs) that contribute to the pathogenic potential and fitness of *C. jejuni* will require a detailed analysis of mutants generated by stress conditions that mimic the host environment.

## Data availability statement

The genome sequences of the C. jejuni isolates determined in this study were deposited in GenBank (Project PRJNA634604, accession: SRR22024544, SRR22024545, SRR22024546, SRR22024547, SRR22024548; SRR22024599, SRR22024600, SRR22024601, SRR22024602, SRR22024603, SRR22024604, SRR22024605, SRR22024606, SRR22024607, and SRR22024608).

## Author contributions

PT, SR, CP, and MK contributed to the conception and design of the study. PT, TC, SH, KT, SL, TH-W, and MK performed experiments. PT and MK organized the data and wrote the first draft of the manuscript. PT performed the statistical analysis. LG and CP wrote sections of the manuscript. All authors contributed to the manuscript revision, read, and approved the submitted version.

## Funding

This research was supported in part by the USDA Agricultural Research Service CRIS project 2030-42000-055-00D and the USDA National Institute of Food and Agriculture project 1017140.

## Conflict of interest

The authors declare that the research was conducted in the absence of any commercial or financial relationships that could be construed as a potential conflict of interest.

## Publisher’s note

All claims expressed in this article are solely those of the authors and do not necessarily represent those of their affiliated organizations, or those of the publisher, the editors and the reviewers. Any product that may be evaluated in this article, or claim that may be made by its manufacturer, is not guaranteed or endorsed by the publisher.

## References

[ref1] Allos B. M. (2001). *Campylobacter jejuni* infections: update on emerging issues and trends. Clin. Infect. Dis. 32, 1201–1206. doi: 10.1086/319760, PMID: 11283810

[ref2] Bae W. Kaya K. N. Hancock D. D. Call D. R. Park Y. H. Besser T. E. (2005). Prevalence and antimicrobial resistance of thermophilic *Campylobacter* spp. from cattle farms in Washington State. Appl. Environ. Microbiol. 71, 169–174. doi: 10.1128/AEM.71.1.169-174.2005, PMID: 15640184 PMC544228

[ref3] Banda D. M. Nunez N. N. Burnside M. A. Bradshaw K. M. David S. S. (2017). Repair of 8-oxoG:a mismatches by the MUTYH glycosylase: mechanism, metals and medicine. Free Radic. Biol. Med. 107, 202–215. doi: 10.1016/j.freeradbiomed.2017.01.008, PMID: 28087410 PMC5457711

[ref4] Barton C. Ng L. K. Tyler S. D. Clark C. G. (2007). Temperate bacteriophages affect pulsed-field gel electrophoresis patterns of *Campylobacter jejuni*. J. Clin. Microbiol. 45, 386–391. doi: 10.1128/JCM.01513-06, PMID: 17135440 PMC1829001

[ref5] Bax B. D. Chan P. F. Eggleston D. S. Fosberry A. Gentry D. R. Gorrec F.. (2010). Type IIA topoisomerase inhibition by a new class of antibacterial agents. Nature 466, 935–940. doi: 10.1038/nature09197, PMID: 20686482

[ref6] Beckmann L. Muller M. Luber P. Schrader C. Bartelt E. Klein G. (2004). Analysis of *gyrA* mutations in quinolone-resistant and -susceptible *Campylobacter jejun*i isolates from retail poultry and human clinical isolates by non-radioactive single-strand conformation polymorphism analysis and DNA sequencing. J. Appl. Microbiol. 96, 1040–1047. doi: 10.1111/j.1365-2672.2004.02242.x, PMID: 15078520

[ref7] Blower T. R. Williamson B. H. Kerns R. J. Berger J. M. (2016). Crystal structure and stability of gyrase-fluoroquinolone cleaved complexes from *Mycobacterium tuberculosis*. Proc. Natl. Acad. Sci. U. S. A. 113, 1706–1713. doi: 10.1073/pnas.1525047113, PMID: 26792525 PMC4763791

[ref8] Bravo V. Katz A. Porte L. Weitzel T. Varela C. Gonzalez-Escalona N.. (2021). Genomic analysis of the diversity, antimicrobial resistance and virulence potential of clinical *Campylobacter jejuni* and *Campylobacter coli* strains from Chile. PLoS Negl. Trop. Dis. 15:e0009207. doi: 10.1371/journal.pntd.0009207, PMID: 33606689 PMC7928456

[ref9] Changkwanyeun R. Yamaguchi T. Kongsoi S. Changkaew K. Yokoyama K. Kim H.. (2016). Impact of mutations in DNA gyrase genes on quinolone resistance in *Campylobacter jejuni*. Drug Test. Anal. 8, 1071–1076. doi: 10.1002/dta.1937, PMID: 26857529

[ref10] Coker A. O. Isokpehi R. D. Thomas B. N. Amisu K. O. Obi C. L. (2002). Human campylobacteriosis in developing countries. Emerg. Infect. Dis. 8, 237–243. doi: 10.3201/eid0803.010233, PMID: 11927019 PMC2732465

[ref11] Cullen T. W. Madsen J. A. Ivanov P. L. Brodbelt J. S. Trent M. S. (2012). Characterization of unique modification of flagellar rod protein FlgG by *Campylobacter jejuni* lipid a phosphoethanolamine transferase, linking bacterial locomotion and antimicrobial peptide resistance. J. Biol. Chem. 287, 3326–3336. doi: 10.1074/jbc.M111.321737, PMID: 22158617 PMC3270987

[ref12] Cullen T. W. Obrien J. P. Hendrixson D. R. Giles D. K. Hobb R. I. Thompson S. A.. (2013). EptC of *Campylobacter jejuni* mediates phenotypes involved in host interactions and virulence. Infect. Immun. 81, 430–440. doi: 10.1128/IAI.01046-12, PMID: 23184526 PMC3553815

[ref13] Dai L. Muraoka W. T. Wu Z. Sahin O. Zhang Q. (2015). A single nucleotide change in *mutY* increases the emergence of antibiotic-resistant *Campylobacter jejuni* mutants. J. Antimicrob. Chemother. 70, 2739–2748. doi: 10.1093/jac/dkv190, PMID: 26169557 PMC4668879

[ref14] Deatherage D. E. Barrick J. E. (2014). Identification of mutations in laboratory-evolved microbes from next-generation sequencing data using breseq. Methods Mol. Biol. 1151, 165–188. doi: 10.1007/978-1-4939-0554-6_12, PMID: 24838886 PMC4239701

[ref15] Demple B. Harrison L. (1994). Repair of oxidative damage to DNA: enzymology and biology. Annu. Rev. Biochem. 63, 915–948. doi: 10.1146/annurev.bi.63.070194.004411 7979257

[ref16] Elliot W. H. (1985). Metabolism of bile acids in liver and extrahepatic tissues, in sterols and bile acids, eds. Danielsson D. Sjovall J.. Elsvier Science Publishers B.V., 303–329. doi: 10.1016/S0167-7306(08)60687-0

[ref17] Engberg J. Aarestrup F. M. Taylor D. E. Gerner-Smidt P. Nachamkin I. (2001). Quinolone and macrolide resistance in *Campylobacter jejuni* and *C. coli*: resistance mechanisms and trends in human isolates. Emerg. Infect. Dis. 7, 24–34. doi: 10.3201/eid0701.010104, PMID: 11266291 PMC2631682

[ref18] Fage C. D. Brown D. B. Boll J. M. Keatinge-Clay A. T. Trent M. S. (2014). Crystallographic study of the phosphoethanolamine transferase EptC required for polymyxin resistance and motility in *Campylobacter jejuni*. Acta Crystallogr. D Biol. Crystallogr. 70, 2730–2739. doi: 10.1107/S1399004714017623, PMID: 25286856 PMC4188012

[ref19] Frenzen P. D. (2008). Economic cost of Guillain-Barré syndrome in the United States. Neurology 71, 21–27. doi: 10.1212/01.wnl.0000316393.54258.d1, PMID: 18591502

[ref20] Gibreel A. Taylor D. E. (2006). Macrolide resistance in *Campylobacter jejuni* and *Campylobacter coli*. J. Antimicrob. Chemother. 58, 243–255. doi: 10.1093/jac/dkl210 16735431

[ref21] Gourley C. R. Negretti N. M. Konkel M. E. (2017). The food-borne pathogen *Campylobacter jejuni* depends on the AddAB DNA repair system to defend against bile in the intestinal environment. Sci. Rep. 7:14777. doi: 10.1038/s41598-017-14646-9, PMID: 29089630 PMC5665897

[ref22] Hakanen A. Jalava J. Kotilainen P. Jousimies-Somer H. Siitonen A. Huovinen P. (2002). *gyrA* polymorphism in *Campylobacter jejuni*: detection of *gyrA* mutations in 162 *C. jejuni* isolates by single-strand conformation polymorphism and DNA sequencing. Antimicrob. Agents Chemother. 46, 2644–2647. doi: 10.1128/AAC.46.8.2644-2647.2002, PMID: 12121947 PMC127378

[ref23] Hakeem M. J. Asseri K. A. Ma L. Chou K. C. Konkel M. E. Lu X. (2019). A novel mathematical model for studying antimicrobial interactions against *Campylobacter jejuni*. Front. Microbiol. 10:1038. doi: 10.3389/fmicb.2019.01038 31139168 PMC6527739

[ref24] Haque M. A. Platts-Mills J. A. Mduma E. Bodhidatta L. Bessong P. Shakoor S.. (2019). Determinants of *Campylobacter* infection and association with growth and enteric inflammation in children under 2 years of age in low-resource settings. Sci. Rep. 9:17124. doi: 10.1038/s41598-019-53533-3, PMID: 31748573 PMC6868199

[ref25] Hawkey P. M. (2003). Mechanisms of quinolone action and microbial response. J. Antimicrob. Chemother. 51, 29–35. doi: 10.1093/jac/dkg207 12702701

[ref26] Hernandez S. B. Cota I. Ducret A. Aussel L. Casadesus J. (2012). Adaptation and preadaptation of *Salmonella enterica* to bile. PLoS Genet. 8:e1002459. doi: 10.1371/journal.pgen.1002459 22275872 PMC3261920

[ref27] Hofmann A. F. Eckmann L. (2006). How bile acids confer gut mucosal protection against bacteria. Proc. Natl. Acad. Sci. U. S. A. 103, 4333–4334. doi: 10.1073/pnas.0600780103, PMID: 16537368 PMC1450168

[ref28] Kakiyama G. Pandak W. M. Gillevet P. M. Hylemon P. B. Heuman D. M. Daita K.. (2013). Modulation of the fecal bile acid profile by gut microbiota in cirrhosis. J. Hepatol. 58, 949–955. doi: 10.1016/j.jhep.2013.01.003, PMID: 23333527 PMC3936319

[ref29] Kearse M. Moir R. Wilson A. Stones-Havas S. Cheung M. Sturrock S.. (2012). Geneious basic: an integrated and extendable desktop software platform for the organization and analysis of sequence data. Bioinformatics 28, 1647–1649. doi: 10.1093/bioinformatics/bts199, PMID: 22543367 PMC3371832

[ref30] Kisker C. Kuper J. Van Houten B. (2013). Prokaryotic nucleotide excision repair. Cold Spring Harb. Perspect. Biol. 5:a012591. doi: 10.1101/cshperspect.a012591 23457260 PMC3578354

[ref31] Krokan H. E. Bjørås M. (2013). Base excision repair. Cold Spring Harb. Perspect. Biol. 5:a012583. doi: 10.1101/cshperspect.a012583 23545420 PMC3683898

[ref32] Lim E. S. Kim J. S. (2017). Role of *eptC* in biofilm formation by *Campylobacter jejuni* NCTC11168 on polystyrene and glass surfaces. J. Microbiol. Biotechnol. 27, 1609–1616. doi: 10.4014/jmb.1610.10046, PMID: 28683522

[ref33] Lin J. Sahin O. Michel L. O. Zhang Q. (2003). Critical role of multidrug efflux pump CmeABC in bile resistance and *in vivo* colonization of *Campylobacter jejuni*. Infect. Immun. 71, 4250–4259. doi: 10.1128/IAI.71.8.4250-4259.2003, PMID: 12874300 PMC165992

[ref34] Luo N. Pereira S. Sahin O. Lin J. Huang S. Michel L.. (2005). Enhanced *in vivo* fitness of fluoroquinolone-resistant *Campylobacter jejuni* in the absence of antibiotic selection pressure. Proc. Natl. Acad. Sci. U. S. A. 102, 541–546. doi: 10.1073/pnas.0408966102, PMID: 15634738 PMC545549

[ref35] Luo N. Sahin O. Lin J. Michel L. O. Zhang Q. (2003). In vivo selection of *Campylobacter* isolates with high levels of fluoroquinolone resistance associated with *gyrA* mutations and the function of the CmeABC efflux pump. Antimicrob. Agents Chemother. 47, 390–394. doi: 10.1128/AAC.47.1.390-394.2003, PMID: 12499221 PMC148968

[ref36] Malik-Kale P. Raphael B. H. Parker C. T. Joens L. A. Klena J. D. Quinones B.. (2007). Characterization of genetically matched isolates of *Campylobacter jejuni* reveals that mutations in genes involved in flagellar biosynthesis alter the organism’s virulence potential. Appl. Environ. Microbiol. 73, 3123–3136. doi: 10.1128/AEM.01399-06, PMID: 17369342 PMC1907099

[ref37] Merritt M. E. Donaldson J. R. (2009). Effect of bile salts on the DNA and membrane integrity of enteric bacteria. J. Med. Microbiol. 58, 1533–1541. doi: 10.1099/jmm.0.014092-0, PMID: 19762477

[ref38] Mixter P. F. Klena J. D. Flom G. A. Siegesmund A. M. Konkel M. E. (2003). In vivo tracking of *Campylobacter jejuni* by using a novel recombinant expressing green fluorescent protein. Appl. Environ. Microbiol. 69, 2864–2874. doi: 10.1128/AEM.69.5.2864-2874.2003, PMID: 12732559 PMC154531

[ref39] Neal-Mckinney J. M. Konkel M. E. (2012). The *Campylobacter jejuni* CiaC virulence protein is secreted from the flagellum and delivered to the cytosol of host cells. Front. Cell. Infect. Microbiol. 2:31. doi: 10.3389/fcimb.2012.00031 22919623 PMC3417660

[ref40] Negretti N. M. Gourley C. R. Clair G. C. Adkins J. N. Konkel M. E. (2017). The food-borne pathogen *Campylobacter jejuni* responds to the bile salt deoxycholate with countermeasures to reactive oxygen species. Sci. Rep. 7:15455. doi: 10.1038/s41598-017-15379-5, PMID: 29133896 PMC5684402

[ref41] Parker C. T. Huynh S. Heikema A. P. Cooper K. K. Miller W. G. (2015). Complete genome sequences of *Campylobacter jejuni* strains RM3196 (233.94) and RM3197 (308.95) isolated from patients with Guillain-Barré syndrome. Genome Announc. 3, e01312–e01315. doi: 10.1128/genomeA.01312-15, PMID: 26543130 PMC4645215

[ref42] Payot S. Bolla J. M. Corcoran D. Fanning S. Megraud F. Zhang Q. (2006). Mechanisms of fluoroquinolone and macrolide resistance in *Campylobacter* spp. Microbes Infect. 8, 1967–1971. doi: 10.1016/j.micinf.2005.12.032, PMID: 16713726

[ref43] Price L. B. Lackey L. G. Vailes R. Silbergeld E. (2007). The persistence of fluoroquinolone-resistant *Campylobacter* in poultry production. Environ. Health Perspect. 115, 1035–1039. doi: 10.1289/ehp.10050, PMID: 17637919 PMC1913601

[ref44] Ridley A. M. Toszeghy M. J. Cawthraw S. A. Wassenaar T. M. Newell D. G. (2008). Genetic instability is associated with changes in the colonization potential of *Campylobacter jejuni* in the avian intestine. J. Appl. Microbiol. 105, 95–104. doi: 10.1111/j.1365-2672.2008.03759.x, PMID: 18298527

[ref45] Ridlon J. M. Kang D. J. Hylemon P. B. (2006). Bile salt biotransformations by human intestinal bacteria. J. Lipid Res. 47, 241–259. doi: 10.1194/jlr.R500013-JLR200 16299351

[ref46] Ruiz-Palacios G. M. (2007). The health burden of *Campylobacter* infection and the impact of antimicrobial resistance: playing chicken. Clin. Infect. Dis. 44, 701–703. doi: 10.1086/509936, PMID: 17278063

[ref47] Sannasiddappa T. H. Lund P. A. Clarke S. R. (2017). *In vitro* antibacterial activity of unconjugated and conjugated bile salts on *Staphylococcus aureus*. Front. Microbiol. 8:1581. doi: 10.3389/fmicb.2017.01581, PMID: 28878747 PMC5572772

[ref48] Scanlan E. Ardill L. Whelan M. V. Shortt C. Nally J. E. Bourke B.. (2017). Relaxation of DNA supercoiling leads to increased invasion of epithelial cells and protein secretion by *Campylobacter jejuni*. Mol. Microbiol. 104, 92–104. doi: 10.1111/mmi.13614, PMID: 28019693 PMC6592826

[ref49] Schwerer B. (2002). Antibodies against gangliosides: a link between preceding infection and immunopathogenesis of Guillain-Barré syndrome. Microbes Infect. 4, 373–384. doi: 10.1016/S1286-4579(02)01550-2, PMID: 11909748

[ref50] Scott N. E. Nothaft H. Edwards A. V. Labbate M. Djordjevic S. P. Larsen M. R.. (2012). Modification of the *Campylobacter jejuni* N-linked glycan by EptC protein-mediated addition of phosphoethanolamine. J. Biol. Chem. 287, 29384–29396. doi: 10.1074/jbc.M112.380212, PMID: 22761430 PMC3436159

[ref51] Shortt C. Scanlan E. Hilliard A. Cotroneo C. E. Bourke B. Ó Cróinín T. (2016). DNA supercoiling regulates the motility of *Campylobacter jejuni* and is altered by growth in the presence of chicken mucus. MBio:e01227-16:7. doi: 10.1128/mBio.01227-16 27624126 PMC5021803

[ref52] Spiller R. C. Jenkins D. Thornley J. P. Hebden J. M. Wright T. Skinner M.. (2000). Increased rectal mucosal enteroendocrine cells, T lymphocytes, and increased gut permeability following acute *Campylobacter* enteritis and in post-dysenteric irritable bowel syndrome. Gut 47, 804–811. doi: 10.1136/gut.47.6.804, PMID: 11076879 PMC1728147

[ref53] Sproston E. L. Wimalarathna H. M. L. Sheppard S. K. (2018). Trends in fluoroquinolone resistance in *Campylobacter*. Microb. Genom 4. doi: 10.1099/mgen.0.000198 PMC6159550 30024366

[ref54] Sung J. Morales W. Kim G. Pokkunuri V. Weitsman S. Rooks E.. (2013). Effect of repeated *Campylobacter jejuni* infection on gut flora and mucosal defense in a rat model of post infectious functional and microbial bowel changes. Neurogastroenterol. Motil. 25, 529–e372. doi: 10.1111/nmo.12118, PMID: 23521493

[ref55] Tacconelli E. Carrara E. Savoldi A. Harbarth S. Mendelson M. Monnet D. L.. (2018). Discovery, research, and development of new antibiotics: the WHO priority list of antibiotic-resistant bacteria and tuberculosis. Lancet Infect. Dis. 18, 318–327. doi: 10.1016/S1473-3099(17)30753-3, PMID: 29276051

[ref56] Talukdar P. K. Negretti N. M. Turner K. L. Konkel M. E. (2020). Molecular dissection of the *Campylobacter jejuni* CadF and FlpA virulence proteins in binding to host cell fibronectin. Microorganisms 8. doi: 10.3390/microorganisms8030389, PMID: 32168837 PMC7143056

[ref57] Talukdar P. K. Turner K. L. Crockett T. M. Lu X. Morris C. F. Konkel M. E. (2021). Inhibitory effect of puroindoline peptides on *Campylobacter jejuni* growth and biofilm formation. Front. Microbiol. 12:702762. doi: 10.3389/fmicb.2021.702762, PMID: 34276635 PMC8283790

[ref58] Urdaneta V. Casadesus J. (2017). Interactions between bacteria and bile salts in the gastrointestinal and hepatobiliary tracts. Front. Med. (Lausanne) 4:163. doi: 10.3389/fmed.2017.00163, PMID: 29043249 PMC5632352

[ref59] Whelan M. V. X. Ardill L. Koide K. Nakajima C. Suzuki Y. Simpson J. C.. (2019). Acquisition of fluoroquinolone resistance leads to increased biofilm formation and pathogenicity in *Campylobacter jejuni*. Sci. Rep. 9:18216. doi: 10.1038/s41598-019-54620-1, PMID: 31796849 PMC6890674

