# Supplementary material for: The bile salt deoxycholate induces Campylobacter jejuni genetic point mutations that promote increased antibiotic resistance and fitness
Source: Front Microbiol. 2022 Dec 21;13:1062464. doi: 10.3389/fmicb.2022.1062464 (PMC9812494; doi:10.3389/fmicb.2022.1062464)

SUPPLEMENTARY MATERIALS

TABLE S1. List of primers used in this study.

Name	Sequence (5' - 3')
**Sequencing primers**	
gyrA-F	ATGATGAGGCAAAAAGTAGAACAG
gyrA-R	TATCAAGCAAATATAAAAGTCCATCTATCAAC
**Mutant construction primers**	
eptC-KO-Up-F	GTACCGGGCCCCCCCTCGACTCGAGATCAGAGCCAAAAATATCATCTC
eptC-KO-Up-R	GGAACACCCGCGGCCCACCTTCAAATTTAAGCTTTTAATTTTATTG
eptC-KO-CAT-F	AGGTGGGCCGCGGGTGTTCCTTTCCAAGTTAA
eptC-KO-CAT-R	TATGCTCCCGCGGGATCTGCGCCCTTTAG
eptC-KO-Dwn-F	GCAGATCCCGCGGGAGCATAAAGGTTTAAAACTTTCTCAAG
eptC-KO-Dwn-F	GGAACAAAAGCTGGGAGCTCCGATGATGGTAGGTTTACTTTGATG
mutY-KO-Up-F	TATAGGGCGAATTGGGTACCGGATTTTAAGTCCCTTGTGTCTACCAATTCCACC
mutY-KO-Up-R	ACACGGATCCAAATGAGAGTATAACAAAAAATTTTAAAATTTTTTGCTAAAATTAGCTC
mutY-KO-CAT-F	ACTCTCATTTGGATCCGTGTTCCTTTCCAAGTTAATTGCG
mutY-KO-CAT-R	GCTTAATTAACTGCAGGATCTGCGCCCTTTAGT
mutY-KO-Dwn-F	GATCCTGCAGTTAATTAAGCTCTGATTTTTTAGGCAAACATAAAGTAGCTATAAGCATG
mutY-KO-Dwn-R	GGGAACAAAAGCTGGAGCTCGGAAATTTAGTAGAGTGGTTTGACTTTTATATCTACGC
uvrC-KO-Up-F	TATAGGGCGAATTGGGTACCCAGGTGCAACTGTGGCTTTGTAATG
uvrC-KO-Up-R	ACACGGATCCTTAGTCAAGATTAGAACCTTTTTTTATAAGTTCTCTTAAATCTTCAAATT
uvrC-KO-CAT-F	TCTTGACTAAGGATCCGTGTTCCTTTCCAAGTTAATTGCG
uvrC-KO-CAT-R	TAATATCAAACTGCAGGATCTGCGCCCTTTAGTTCC
uvrC-KO-Dwn-F	GATCCTGCAGTTTGATATTAGCAGGGATAATTTTTATTTTTAATCTAATTTTAGCGTATT
uvrC-KO-Dwn-R	GGGAACAAAAGCTGGAGCTCTGCTTCATTTGCGCTCACAAAGC
**Complementation primers**	
eptC-Comp-F	GTTGGATCACCTCCTTTCTAGAGCTCTTAAGCCATTGACATTCC
eptC-Comp-R	CTTGGGCAAGAGCTTTGGATCCCCTTCTAAAGCATATCTTGCG
mutY-Comp-F	CACCTCCTTTCTAGAACTATTGTCTAATGTGATTTTTGAGCTTAAATTTTCATTTGC
mutY-Comp-R	GAGCTTTGAATTCGGATCcTTATTTATCATCATCATCTTTATAATCAATATCATGATCTT TATAATCACCATCATGATCTTTATAATCGAGCTTAATTAATTTTAACGCTTTTAAAGAAA GTGC

FIGURE S1. Quinolone resistance-determining region (QRDR). The quinolone resistance-determining region is between the 69th and 120th codon of the *gyrA* gene, which codes for *C. jejuni* DNA gyrase. The two codons of interest where a point mutation may confer ciprofloxacin resistance are 86 and 90, coding for threonine and aspartate, respectively.

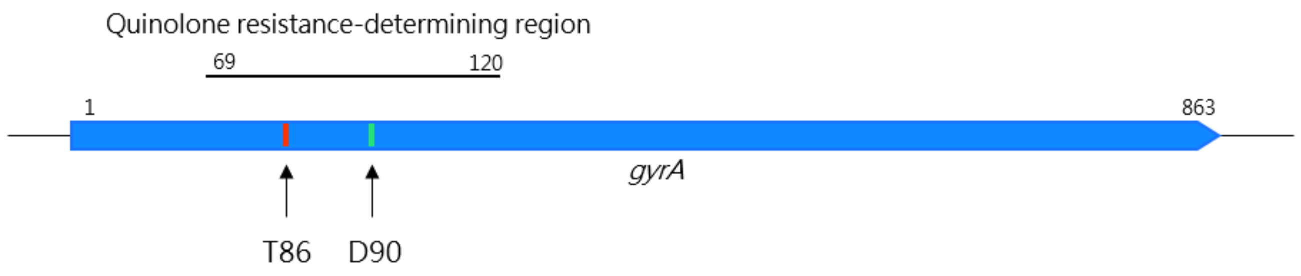

FIGURE S2. Growth of the *C. jejuni* 81-176 strain and select *C. jejuni* 81-176 ciprofloxacin mutants in MH broth, as determined by optical density (OD_{540}). The 81-176 gyrA 86 ATA 101 and 81-176 gyrA 86 ATA 105 isolates contain a mutation at residue 86. The 81-176 gyrA 90 ATA 110 and 81-176 gyrA 90 ATA 113 isolates contain a mutation at residue 90. No difference in the growth of *C. jejuni* mutants was observed compared to the wild-type strain. The data are from a single experiment and are representative of three independent assays (technical replicates).

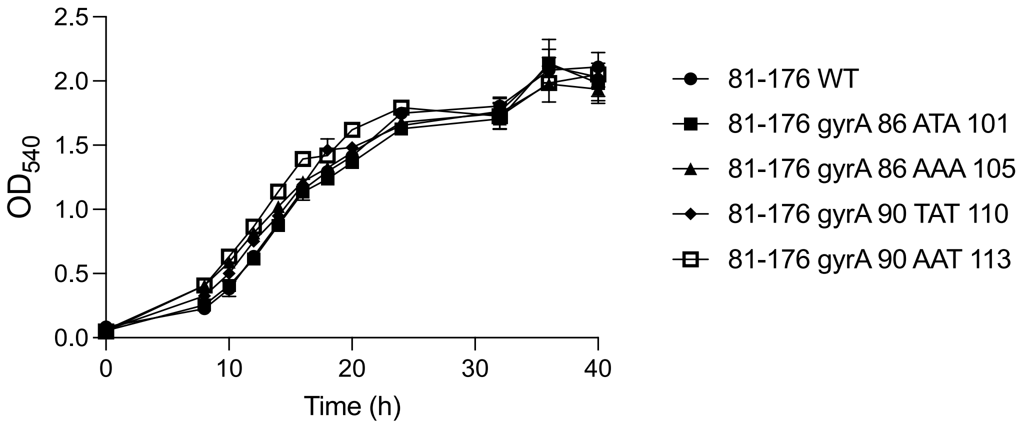

FIGURE S3. The optical density (OD_{540}) of the individual *C. jejuni* variants. *C. jejuni* variants passaged in deoxycholate (DOC) show enhanced growth in DOC-supplemented media. Select *C. jejuni* isolates were picked and cultured in MH medium or MH medium supplemented with 0.1% and 0.4% sodium deoxycholate. The cultures were grown in a microaerobic chamber at 37°C with shaking for 24 hours.

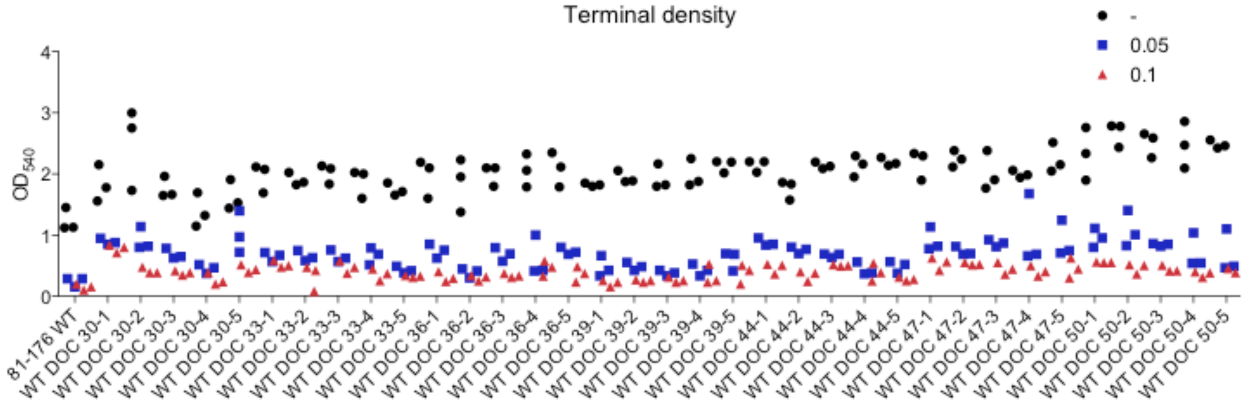

FIGURE S4. Nucleotide sequence and deduced amino acid sequence of the *C. jejuni* 81-176 *eptC* gene (CJJ81176_RS01240). Putative −10 (TATTTT) and −35 (TTGATC) promoter elements are overlined. The gene is 1784 bp and encodes a protein of 59.4 kDa. The deduced amino acid sequence is indicated below the *eptC* gene sequence in a single-letter code. The in-frame translational stop codon is marked with an asterisk. Marked is the position of the P360Q mutation.

```
                   -35                                 -10
TCTAGAGCTCTTAAGCCATTGACATTCCATGAAAGTAGTTTCATTATTTTCCTTAAAATTTTCATTAAAAGTGTAAATCATAACAT

AAAAAGATTTAAAACTACAATCCATTTAAAAAAATTCAATAAAATTAAAAGCTTAAATTTGAAGGTGGGATTTATATGCTTAGATTA
                                                                             M  L  R  L

ACTTGGTTTCAGTTTACTTTTTTTAATTCTTTGATGATTGTATTATTGAATTTTAATTTATTTTATTTTGTGTATGAAAAAAATACA
 T  W  F  Q  F  T  F  F  N  S  L  M  I  V  L  L  N  F  N  L  F  Y  F  V  Y  E  K  N  T

CAAAATTGGTTTATAACTTTTGTTTTTATTGTGGCCTATTTTGCACTTGTTCACGTGATTTGCTCTTTGCTTTTTATAAAGTTTTTT
 Q  N  W  F  I  T  F  V  F  I  V  A  Y  F  A  L  V  H  V  I  C  S  L  L  F  I  K  F  F

ACTAAATTTTTTTCTATATTATTTATAATATCATCTTTTTTAAGTGTTTACTTTATTAGTTTTTATGGGGTGCTTATAGATTCTGAT
 T  K  F  F  S  I  L  F  I  I  S  S  F  L  S  V  Y  F  I  S  F  Y  G  V  L  I  D  S  D

ATGATACAAAATGTTGTTCAAACAGACATTAAAGAAGTAAAAGATTTGTTAAATTTAAAATTGATCTTATTTATTGTTTTGGCTTTA
 M  I  Q  N  V  V  Q  T  D  I  K  E  V  K  D  L  L  N  L  K  L  I  L  F  I  V  L  A  L

TTATTGGTTTTTTATGTTGTTAAAGTAAAAATTGATTATTATGGTAGTTTTAAATCCCATATAAAAATTAAAATTATCAATATAATA
 L  L  V  F  Y  V  V  K  V  K  I  D  Y  Y  G  S  F  K  S  H  I  K  I  K  I  I  N  I  I

TCAGGTTTGATTGTTGTTTGTGCAGTTTTAATCCCTTTAAGTAAAACTTTCTTGCCTTTTTTTAGAAACTATAATGAAATAAGAATG
 S  G  L  I  V  V  C  A  V  L  I  P  L  S  K  T  F  L  P  F  F  R  N  Y  N  E  I  R  M

TATAATACTCCTTTTTATCAAATTTATGCCGTATATCGCTATTATGTTCGTTTTGTGAAAGCTAAGCCAGAATTTAAAACCATAGCT
 Y  N  T  P  F  Y  Q  I  Y  A  V  Y  R  Y  Y  V  R  F  V  K  A  K  P  E  F  K  T  I  A

AATGATGCTTATAGAGAAAACAATCACACTAAAAAACTTTTAGTTTTGGTAGTGGGTGAAACCGCTAGAGCGGCAAACTACTCTTTG
 N  D  A  Y  R  E  N  N  H  T  K  K  L  L  V  L  V  V  G  E  T  A  R  A  A  N  Y  S  L

GGTGGATATACTAAAAATGATACCAATTTTTATACCAAAAAAGATAATGTAGTCTTTTTTGATAATTTTTCATCATGTGGAACAGCA
 G  G  Y  T  K  N  D  T  N  F  Y  T  K  K  D  N  V  V  F  F  D  N  F  S  S  C  G  T  A

ACAGCGGTAAGTTTACCTTGTATGTTTTCTATTTCTAAGCGTGAAAATTATTCAAGTTCAGAATTTCAAGAAAATGCGATGGATGTG
 T  A  V  S  L  P  C  M  F  S  I  S  K  R  E  N  Y  S  S  S  E  F  Q  E  N  A  M  D  V

CTTTATAAAACAGGTGTAGATGCTGCATGGTTTGATAATAATTCTGGTGGTTGTAAAGGGGTTTGTGATAGATTAGCTTATAAGCAA
 L  Y  K  T  G  V  D  A  A  W  F  D  N  N  S  G  G  C  K  G  V  C  D  R  L  A  Y  K  Q

AAGCTTTCTAGTGATTTGGATGAAAATTTACTCATTCCTTTTAAAGAAAAATTAAATCATTTAAGTGATCAAAATATAATAGTTCTT
 K  L  S  S  D  L  D  E  N  L  L  I  P  F  K  E  K  L  N  H  L  S  D  Q  N  I  I  V  L

CATTTGCAAGGTTCTCATGGGCCAACTTATTATAAACGCTATCCAAGTGAATTTAAAAAATTTACTCCAACTTGTGATACTAATGAA
 H  L  Q  G  S  H  G  P  T  Y  Y  K  R  Y  P  S  E  F  K  K  F  T  P  T  C  D  T  N  E

CTTTCAAAATGTGATAGCGAGGCTTTAATCAACACTTATGACAATACTTTGCTTTATACAGATTATCTTTTAAGTGAGATTATAAAA
 L  S  K  C  D  S  E  A  L  I  N  T  Y  D  N  T  L  L  Y  T  D  Y  L  L  S  E  I  I  K

CTATTAAAAGAGCAAAAAAGCTATGAAAGTTCTTTGTTTTATCTTTCAGATCATGGCGAAAGTTTGGGTGAAAATGGTATTTATCTT
 L  L  K  E  Q  K  S  Y  E  S  S  L  F  Y  L  S  D  H  G  E  S  L  G  E  N  G  I  Y  L

CATGGTATGCCTTATGCTATAGCGCCAAGTTATCAAACTCATATTCCTGCTATTTTTTGGAGCAATGATGAAAAATTAATGAATTTA
 H  G  M  P  Y  A  I  A  P  S  Y  Q  T  H  I  P  A  I  F  W  S  N  D  E  K  L  M  N  L

GCTAAAGAGCATAAAGGTTTAAAACTTTCTCAAGATAATCTTTTTAGCACTCTTTTAGGATATTTTAATGTAAAAACAAGTGTTTAT
 A  K  E  H  K  G  L  K  L  S  Q  D  N  L  F  S  T  L  L  G  Y  F  N  V  K  T  S  V  Y

GAGCCAGAATATGATTTATTAAATCCTAAACTTAAGGCAAATCCATGAAACCTAAATATCATTTTTTAAATAACGCAAGATATGCTTT
 E  P  E  Y  D  L  L  N  P  K  L  K  A  N  P  *

AGAAGGGGATCC
```

FIGURE S5. A *C. jejuni* △*eptC* mutant has reduced motility. Motility assays of a *C. jejuni* wild-type (WT) isolate, △*eptC* mutant, △*flgL* mutant, and △*eptC* mutants complemented with either the $eptC_{WT}$ protein (△*eptC* + $eptC_{WT}$) or *eptC* P360Q variant (△*eptC* + $eptC_{WT(P360Q)}$). *C. jejuni* strains were cultured on MH 0.4% agar plates for 48 hours. The values shown in the table are the mean ± standard deviation (SD) of the distances from the edge of the culture spot to the haze of motility from at least three biological replicates. One representative image is shown. *P*-values were calculated with the non-parametric Mann-Whitney *U* test using GraphPad Prism 9.0. *P*-values indicate whether the zone of motility of the isolate was significant (P <0.05) or not (P >0.05) compared to the WT motility.

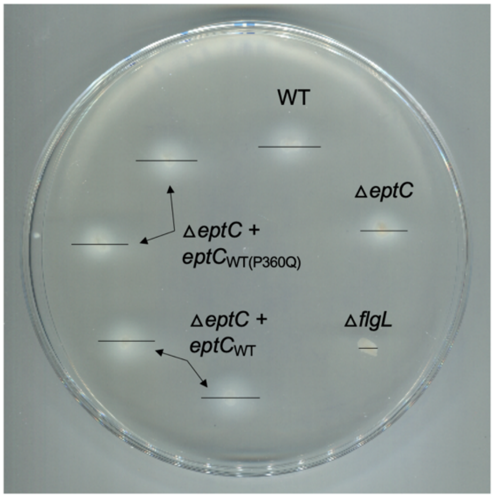

Isolate	Zone (mm)	*P*-value
Wild-type	31.7 ± 1.3	–
△*eptC*	19.6 ± 3.8	0.0030
△*flgL*	5.3 ± 1.1	0.0061
△*eptC* + $eptC_{WT}$	26.8 ± 1.5	0.0095
△*eptC* + $eptC_{WT(P360Q)}$	29.3 ± 0.6	0.0875

FIGURE S6. The growth and ciprofloxacin mutation frequency of *C. jejuni* Δ*uvrC* and Δ*addAB* mutants with and without 0.1% deoxycholate (DOC). Growth of the *C. jejuni* 81-176 A) wild-type, B) Δ*uvrC* mutant, and C) Δ*addAB* mutant in MH or MH supplemented with 0.1% DOC for 36 h and OD_{540} was observed at different time points. Ciprofloxacin mutation frequency of the *C. jejuni* D) wild-type, E) Δ*uvrC* mutant, and F) Δ*addAB* mutant serially passaged for 10 days with or without 0.1% DOC and plated to determine ciprofloxacin mutation frequency. Three biological replicates and the standard error of the mean are shown.

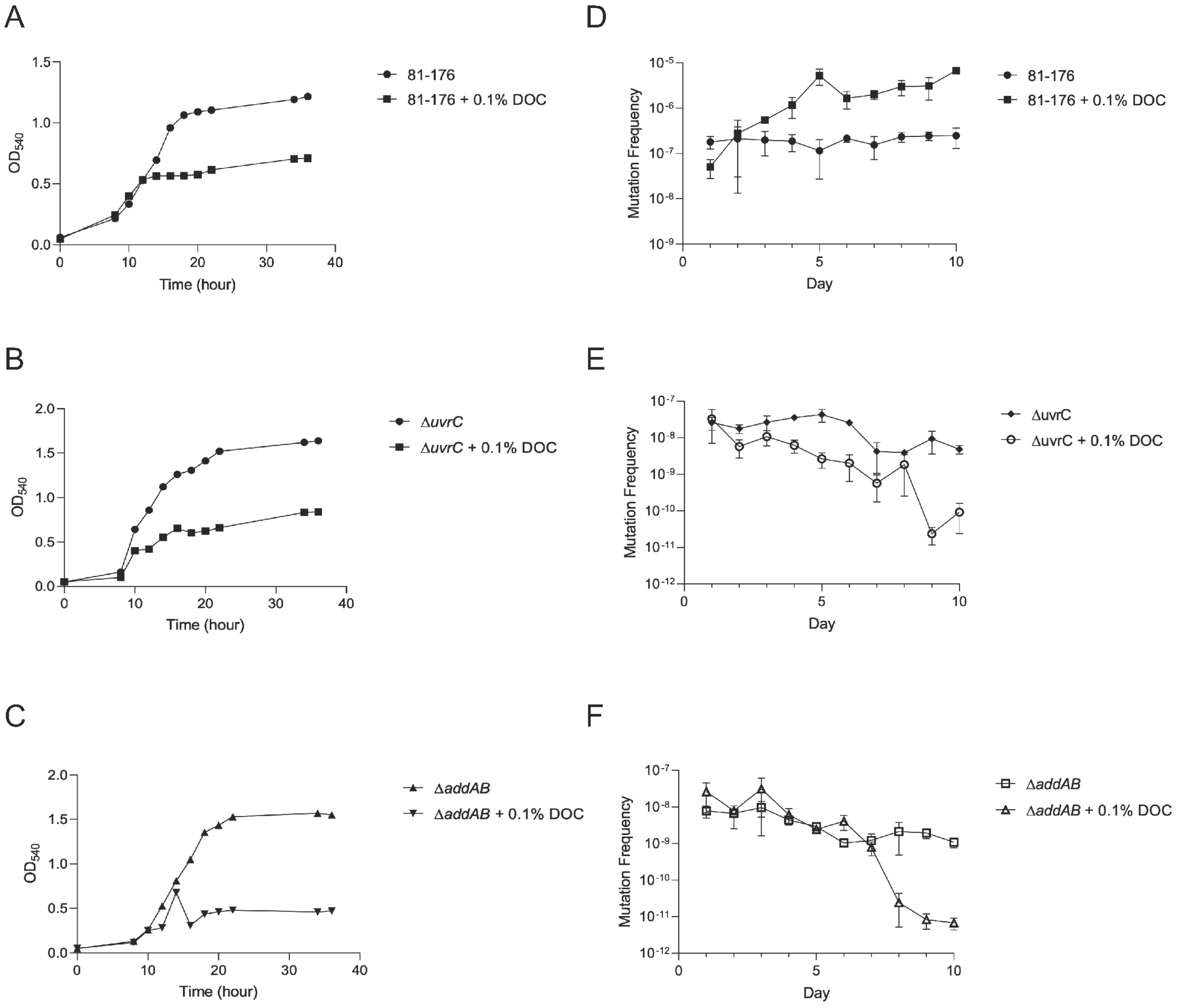

FIGURE S7. Mutation of Thr-86 and Asp-90 in the *C. jejuni* GyrA protein confers increased resistance to ciprofloxacin. The residues equivalent to Thr-86 and Asp-90 in the *C. jejuni* GyrA protein are highlighted in the structure of *Staphylococcus aureus* GyrA, which has Ser and Glu, respectively, at these positions. The Ser/Thr-86 sidechain forms a conserved hydrogen bond to the carboxylate of ciprofloxacin. Mutation of residue 86 to Ile or Lys would result in loss of GyrA-ciprofloxacin interaction and potentially cause steric clashes in the surrounding contacts with ciprofloxacin and DNA termini. The Glu/Asp-90 contributes negative charge to the local environment of the Mn^{2+} ion chelated by the carboxylate and hydroxyl groups of ciprofloxacin. Mutation of residue 90 to Tyr or Asn would disrupt these electrostatic interactions. The figure was generated from the X-ray crystal structure of the *S. aureus* GyrA protein (2XCT pdb). For clarity, the remainder of the third DNA molecule is truncated.

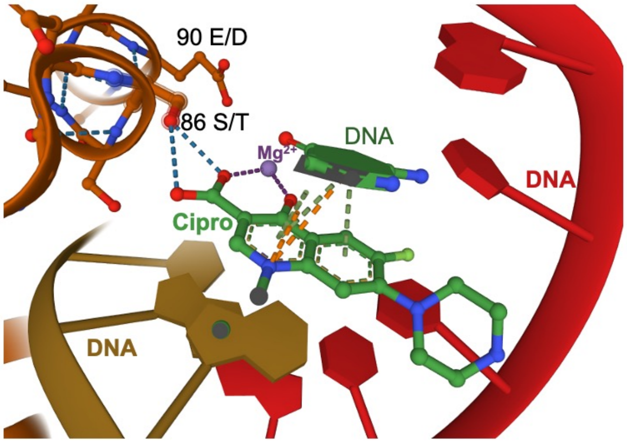

Supplement: Supplementary file 1 [file Data_Sheet_1.PDF]
